# Habitat‐based biodiversity assessment for ecosystem accounting in the Murray–Darling Basin

**DOI:** 10.1111/cobi.13915

**Published:** 2022-05-27

**Authors:** Karel Mokany, Chris Ware, Thomas D. Harwood, Rebecca K. Schmidt, Simon Ferrier

**Affiliations:** ^1^ CSIRO Canberra Australian Capital Territory Australia; ^2^ CSIRO Sandy Bay Tasmania Australia

**Keywords:** Australia, ecosystem accounts, generalized dissimilarity modeling, plants, SEEA EA, threatened species, waterbirds, Australia, aves acuáticas, contabilidad de los ecosistemas, especies amenazadas, modelo de disparidad generalizada, plantas, SEEA EA, 生态系统核算, 澳大利亚, 广义相异性模型, 植物, 环境经济核算体系‐生态系统核算(SEEA EA), 受威胁物种, 水鸟

## Abstract

Understanding how biodiversity is changing over space and time is crucial for well‐informed decisions that help retain Earth's biological heritage over the long term. Tracking changes in biodiversity through ecosystem accounting provides this important information in a systematic way and readily enables linking to other relevant environmental and economic data to provide an integrated perspective. We derived biodiversity accounts for the Murray–Darling Basin, Australia's largest catchment. We assessed biodiversity change from 2010 to 2015 for all vascular plants, all waterbirds, and 10 focal species. We applied a scalable habitat‐based assessment approach that combined expected patterns in the distribution of biodiversity from spatial biodiversity models with a time series of spatially complete data on habitat condition derived from remote sensing. Changes in biodiversity from 2010 to 2015 varied across regions and biodiversity features. For the entire Murray–Darling Basin, the expected persistence of vascular plants increased slightly from 2010 to 2015 (from 86.8% to 87.1%), mean species richness of waterbirds decreased slightly (from 12.5 to 12.3 species), whereas for the focal species the estimated area of habitat increased for 8 species and decreased for 1 species. Regions in the north of the Murray–Darling Basin generally had decreases in biodiversity from 2010 to 2015, whereas in the south biodiversity was stable or increased. Our results demonstrate the benefits of habitat‐based biodiversity assessments in providing fully scalable biodiversity accounts across different biodiversity features, consistent with the United Nations System of Environmental Economic Accounting – Ecosystem Accounting (SEEA EA) framework.

## Abstract (es)

Evaluación de la Biodiversidad con base en el Hábitat para la Contabilización de Ecosistemas en la Cuenca Murray‐Darling

**Resumen:**

El conocimiento sobre cómo está cambiando la biodiversidad en el tiempo y en el espacio es crucial para las decisiones bien informadas que ayudan a retener la herencia biológica de la Tierra a largo plazo. El seguimiento de cambios en la biodiversidad mediante la contabilidad de los ecosistemas proporciona esta información importante de manera sistémica y permite fácilmente la conexión con otros datos ambientales y económicos relevantes para proporcionar una perspectiva integrada. Derivamos la contabilidad de la biodiversidad para la Cuenca Murray‐Darling, la mayor cuenca de Australia. Analizamos los cambios en la biodiversidad entre 2010 y 2015 de todas las plantas vasculares, todas las aves acuáticas y diez especies focales. Aplicamos una estrategia de evaluación basada en el hábitat que combinó los patrones esperados en la distribución de la biodiversidad a partir de modelos espaciales de la biodiversidad con una serie temporal de datos espacialmente completos derivados de la teledetección de la condición del hábitat. Los cambios en la biodiversidad entre 2010 y 2015 variaron entre las regiones y las características de la biodiversidad. Para toda la Cuenca Murray‐Darling, la persistencia esperada de las plantas vasculares incrementó ligeramente durante los años de estudio (de 86.8% a 87.1%), la riqueza promedio de especies de aves acuáticas disminuyó un poco (de 12.5 a 12.3 especies), mientras que el área estimada del hábitat de las especies focales incrementó para ocho especies y disminuyó para una. Las regiones al norte de la cuenca tuvieron disminuciones generalizadas de la biodiversidad entre 2010 y 2015, mientras al sur, la biodiversidad se mantuvo estable o incrementó. Nuestros resultados demuestran los beneficios que tienen las evaluaciones de la biodiversidad basadas en el hábitat para proporcionar una contabilidad de la biodiversidad completamente escalable entre las diferentes características de la biodiversidad, acorde con la estructura del Sistema de Contabilidad Económico‐Ambiental – Contabilidad de los Ecosistemas (SEEA EA) de las Naciones Unidas.

## 【摘要】

了解生物多样性的时空变化对于制定帮助地球生物遗产长期续存的决策至关重要。通过生态系统核算追踪生物多样性的变化, 可以系统地提供这一重要信息, 并能随时与其他相关的环境和经济数据相联系, 提供综合性视角。本研究对澳大利亚最大的集水区——墨累达令盆地的生物多样性进行了核算, 评估了2010年到2015年所有维管植物、所有水鸟和10个重点保护物种的生物多样性变化。我们采用了一种尺度可变的基于栖息地的评估方法, 整合了空间生物多样性模型的生物多样性分布格局估计与遥感得到的栖息地状况空间完整数据的时间序列。研究发现, 2010年至2015年, 生物多样性的变化在不同地区和生物多样性特征之间有所差异。整个墨累达令流域维管植物的预期续存情况在2010年到2015年略有增加(从86.8%到87.1%), 水鸟的平均物种丰富度略有下降(从12.5种到12.3种), 而重点保护物种中8个物种的栖息地面积预计增加, 1个物种的栖息地面积下降。此外, 墨累达令盆地北部地区的生物多样性从2010年到2015年普遍下降, 而南部地区的生物多样性则保持稳定或有所增长。我们的结果表明, 基于栖息地的生物多样性评估有助于提供不同生物多样性特征全尺度的生物多样性核算, 这也符合联合国环境经济核算体系‐生态系统核算(SEEA EA)框架。**【翻译:胡怡思;审校:聂永刚】**

## INTRODUCTION

Understanding how biodiversity is changing over space and time is crucial to making well‐informed decisions that help retain Earth's biological heritage. Given the escalating species extinction crisis (IPBES, 2019), reliable information on where and when biodiversity decline is most severe is increasingly needed to plan smart conservation interventions. Similar information is needed to track the effectiveness of recent policy, planning, and management decisions in reversing declines and promoting biodiversity persistence.

In responding to these needs, a range of biodiversity monitoring and reporting initiatives have been implemented around the world. These initiatives notably include reporting under the Convention on Biological Diversity (CBD), such as the Global Biodiversity Outlook (Secretariat of the Convention on Biological Diversity, 2020), regional and global assessments under the Intergovernmental Platform on Biodiversity and Ecosystem Services (IPBES) (IPBES, 2019), and various programs led by the International Union for the Conservation of Nature (IUCN) (e.g., IUCN Red List of Species [IUCN, 2021; Szabo et al., 2012], IUCN Red List of Ecosystems [Bland et al., 2019], World Database on Protected Areas [Bingham et al., 2019], and Key Biodiversity Areas [IUCN, 2016]).

Such reporting initiatives provide a solid foundation of information on which to base biodiversity‐focused policy and management decisions. However, there is a further need to regularly connect standardized biodiversity information with macrolevel economic and social decision‐making and reporting through an integrated systems approach (Vardon et al., 2019). Accounting for biodiversity across the same spatial jurisdictions and temporal frequencies as economic accounts meets this need. It enables more direct linking of information on biodiversity change to the decision‐making processes that will influence future changes, through the consequences of economic decisions for natural systems (King et al., 2021).

The United Nations Statistics Division has been leading the development of the System of Environmental‐Economic Accounting—Ecosystem Accounting (SEEA EA) framework to standardize the quantification of stocks of ecosystem assets, including biodiversity and flows of ecosystem services over time (UNCEEA, 2021). The SEEA EA is a spatially explicit, integrated statistical framework for organizing biophysical information about ecosystems and ecosystem services. A main focus for the SEEA EA is quantifying over an accounting period the extent and condition of different ecosystem types (considered as separate assets), as well as the ecosystem services that flow from these, and then linking this information to measures of economic and human activity (UNCEEA, 2021). Importantly, the SEEA EA aims to provide information that is comprehensive, structured, consistent, coherent, and spatially referenced (UNCEEA, 2021), ensuring suitability to inform decision‐making at multiple scales.

In the SEEA EA, biodiversity is considered under a “thematic account” that is additional to the core ecosystem accounts (UNCEEA, 2021). The SEEA EA recognizes the multiple dimensions of biodiversity (genetic, species, ecosystem) and provides general principles for accounting for biodiversity, rather than strict guidance on which types of data should be used and how they should be analyzed. According to the general principles of SEEA EA, thematic accounting for biodiversity should involve a clearly defined ecosystem accounting area, the geographical territory for which an ecosystem account is compiled; a defined set of entities that are the focus of accounting; and the derivation of multiple accounts within a theme, in this case biodiversity, to organize relevant information (UNCEEA, 2021). The SEEA EA provides an example species account (UNCEEA, 2021), but no examples of accounts that consider other dimensions of biodiversity, such as community‐level diversity.

To populate biodiversity accounts under the SEEA EA, the most rigorous data source is field observations of species occurrences, population abundances, and community diversity, such as those acquired through ecological monitoring surveys. While these observations provide direct indications of the status of species and communities (Remme et al., 2016; UNEP‐WCMC, 2016), they present challenges for use in SEEA EA accounts. Field observations are typically undertaken at a limited number of locations and times, with the locations, timing, effort, and methods employed often varying over time (Buckland et al., 2012). Surveys are also often biased in terms of the species, assemblages, and ecosystems they focus on (UNEP‐WCMC, 2019). For the few taxa for which suitably comprehensive, consistent observation data exist, such as birds, accounting based on these data typically involves model‐based inference and is better suited to larger jurisdictions (Collen et al., 2009; Hein et al., 2020; van Strien et al., 2016).

**FIGURE 1:**
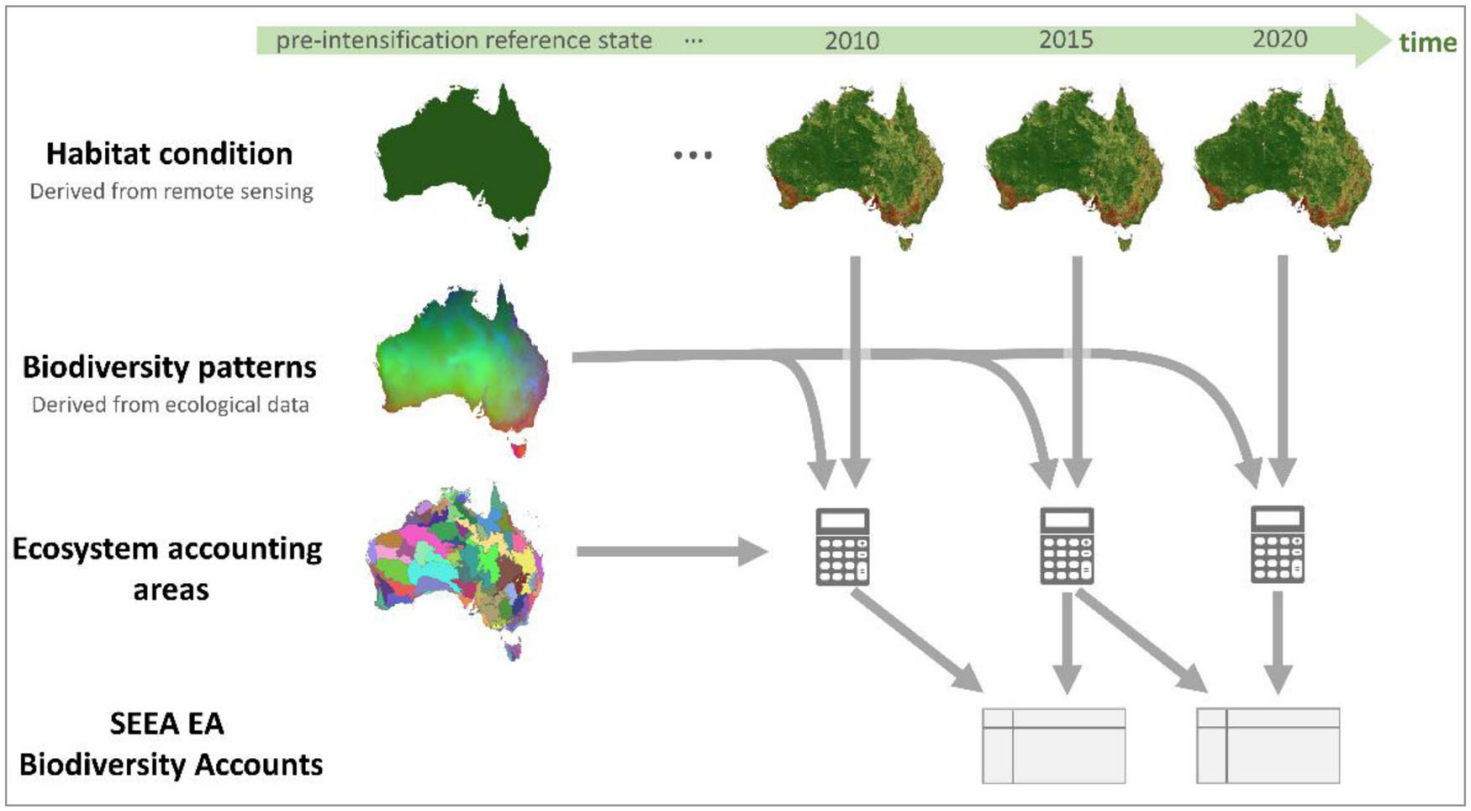
Conceptual depiction of the habitat‐based approach to assessment and accounting for biodiversity. Changes in habitat condition over time can be combined with information on biodiversity patterns, allowing reporting for any ecosystem accounting areas

Habitat‐based assessments can be a valuable tool in accounting for biodiversity, complementing approaches based on direct observations. Habitat‐based approaches rely on observing changes in the spatial extent, quality, and configuration of a species’ habitat (King et al., 2021). Many of these approaches estimate habitat condition based on remotely sensed imagery, harnessing the benefits of remotely sensed data (i.e., standardized, regular, fine resolution, comprehensive extent, low cost). Habitat information derived from remote sensing can be combined with estimated spatial patterns in biodiversity to assess the implications of changes in habitat on biodiversity features of interest (UNEP‐WCMC, 2016) (Figure 1). One of the key benefits of habitat‐based biodiversity assessments for SEEA EA accounting is that they are highly scalable, enabling consistent and comparable reporting for any area or jurisdiction over time (Bond et al., 2013; Ivanov et al., 2013; Mokany et al., 2019).

**FIGURE 2:**
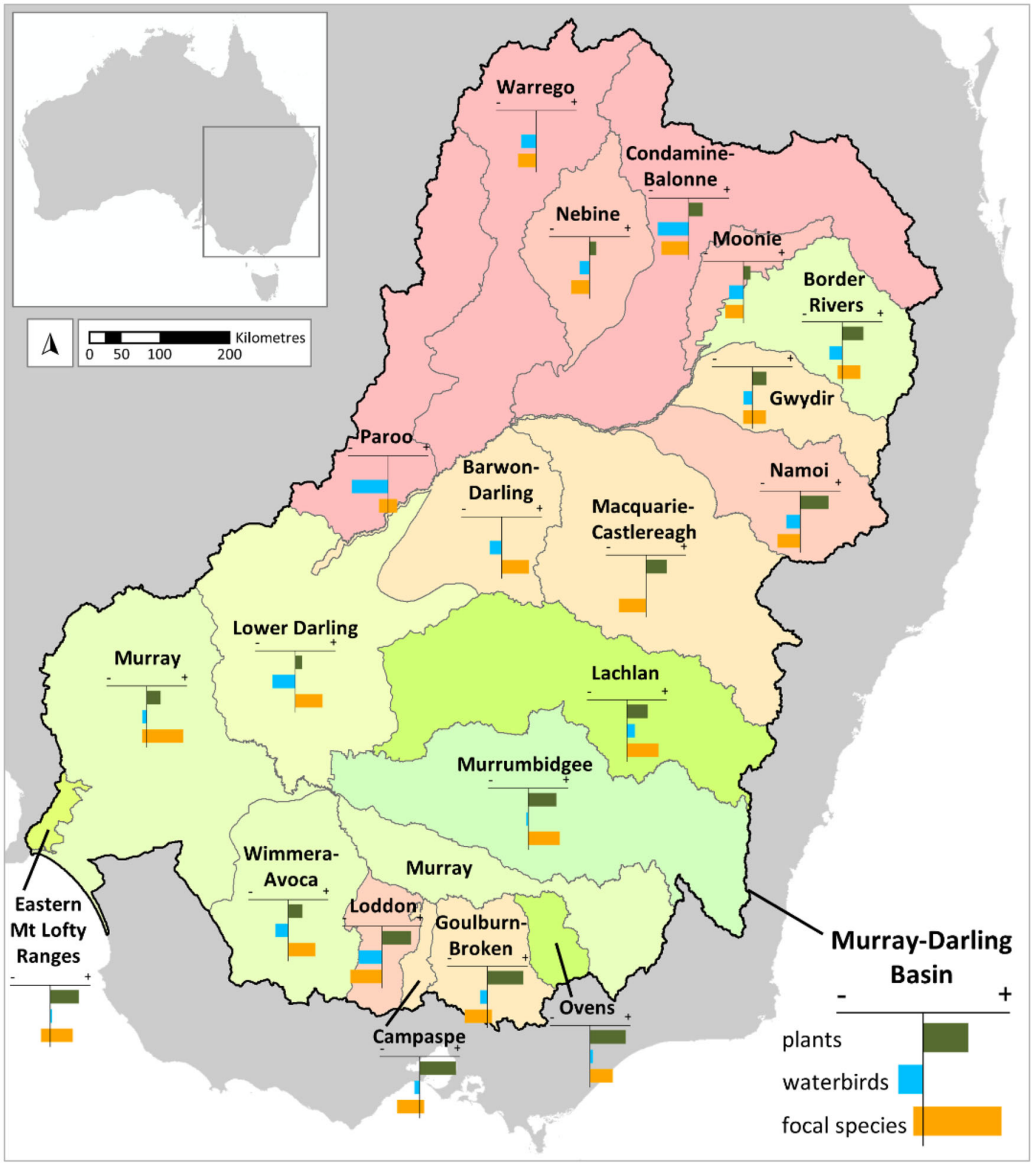
Summary of the biodiversity accounting results (i.e., changes in vascular plants, waterbirds, and focal species) across the regions of the Murray–Darling Basin (MDBA, 2014) in southeastern Australia. For each region, the color represents the combined change across biodiversity features from 2010 to 2015 (red = decrease, green = increase, darker = greater change). Bar graphs in each region and for the entire Murray–Darling Basin show the relative change in each biodiversity feature from 2010 to 2015, reported on a common scale to enable comparison between regions. For plants, the measure is percentage of species expected to persist over the long term. For waterbirds, the measure is average number of species expected. For the focal species, the measure is number of species whose area of habitat increases or decreases over time

We applied a habitat‐based approach to accounting for biodiversity in the Murray–Darling Basin in Australia for 2010 and 2015, which align with experimental land accounts for Australia (ABS, 2021). The Murray–Darling Basin is Australia's largest catchment, covering 1.06 million km^2^. It generates $AU24 billion of agricultural revenue per year (approximately 40% of Australia's total agricultural revenue), including significant irrigated agriculture from diverted water flows (MDBA, 2020). To assess biodiversity change, we used habitat‐based accounts developed using a number of approaches to accommodate complementary facets of biodiversity, including species‐level accounts for 10 focal species and community‐level accounts for vascular plants and waterbirds. We sought to provide a fine‐resolution spatially explicit assessment that would allow reporting across scales, including the whole Murray–Darling Basin and regions within the basin used for monitoring and management (MDBA, 2014) (Figure 2). We also aimed to provide a method that can be used to inform the ongoing development of guidance on accounting for biodiversity (Section 13.17 in UNCEEA [2021]).

## METHODS

### Broad approach to biodiversity assessment

The biodiversity assessment and accounting method we applied combines expected patterns in the distribution of biodiversity from spatial biodiversity models with a time series of spatially complete data on habitat condition derived from remote sensing (Ferrier et al., 2017) (Figure 1; UNEP‐WCMC, 2016). Expected patterns of biodiversity are those likely to occur in the absence of human land use (excluding Indigenous uses). Human land use over time influences habitat, which then influences expected biodiversity persistence into the future. This habitat‐based approach can incorporate large amounts of ecological survey data in deriving the spatial biodiversity models, ensuring well‐informed estimates of biodiversity patterns.

### Community‐level biodiversity assessment for vascular plants

For vascular plants, the percentage of species expected to persist over the long term (*P*γ) in the ecosystem accounting area, given changes in habitat condition, was determined using the approach described by Ferrier et al. (2004) and subsequently applied in a range of studies (e.g., Allnutt et al., 2008; Di Marco et al., 2019; UNEP‐WCMC, 2016).

This community‐level *P*γ approach (Appendix S1) has 4 main inputs. First, a spatial habitat condition layer for each time point of interest is required. We applied the habitat condition across Australia for 2010 and 2015, as estimated by the Australian Habitat Condition Assessment System (HCAS) 2.1 (Harwood et al., 2021; 2021; Williams et al., 2021) (Appendix S2). Second, spatial layers from a generalized dissimilarity model (GDM) (Ferrier et al., 2007) are used to estimate the level of species assemblage similarity between pairs of locations expected if these locations were still in reference condition (i.e., they exhibited the highest possible level of ecological integrity). We derived a GDM for vascular plants with data from 66,608 survey plots and projected at 90‐m resolution across southeastern Australia (Appendix S3). Third, a spatial layer of expected species richness under reference habitat conditions can be included in estimating *P*γ. We derived a species richness model for vascular plants with data from 66,608 survey plots and projected species richness at 90‐m resolution across southeastern Australia (Appendix S3). Fouth, a spatially defined analysis region is required. We defined the ecosystem accounting areas as the Murray–Darling Basin and the regions within it (Figure 2) (MDBA, 2014).

The community‐level biodiversity assessment for vascular plants was undertaken at 3 arcsecond (≈90 m) spatial resolution across the New South Wales and Australian Capital Territory Regional Climate Model (NARCLiM) region of southeastern Australia (Evans et al., 2014), which covers the entire Murray–Darling Basin. The community‐level *P*γ analyses for each ecosystem accounting area were implemented using CSIRO's BILBI biodiversity assessment system in a customised Python environment.

The nonadditive nature of the species persistence analysis applied to vascular plants means that it considers the benefits of improvements in habitat condition in other locations that support a similar assemblage of species. In the present case, improved habitat condition in 1 location can promote the persistence of the species occurring there, which subsequently influences species persistence across all the other locations where those species occur (Allnutt et al., 2008; Mokany et al., 2019).

### Community‐level biodiversity assessment for waterbirds

For waterbird species assemblages, the biodiversity assessment focused on predicted changes in patterns of species richness over time (Appendix S4), with accounts reporting differences in average species richness between years and between ecosystem accounting areas. Although the original intent was to implement the same analytical approach as that used for vascular plant persistence, this was not possible due to poor predictive performance of fitted GDMs in explaining waterbird species assemblage turnover. Waterbird species richness was derived from 229,162 field observations and modeled as a function of water observations from space (WOfS) (Mueller et al., 2016) and a range of additional static environmental predictors (Appendix S3). The waterbird species richness model was then projected for 2010 and 2015 based on the WOfS data for those years. The resulting maps of predicted species richness for waterbirds provided the information needed to assess mean species richness in each ecosystem accounting area for the 2 years (2010 and 2015).

### Species‐level biodiversity assessment

A habitat‐based approach was also used in accounting for a number of species. The approach combined remote sensing of land‐cover attributes with best‐available mapping of the original spatial distribution of each focal species (Soberón & Peterson, 2009; Tracewski et al., 2016; UNEP‐WCMC, 2016). Ten focal species were selected, representating different taxonomic groups and threat status across the Murray–Darling Basin (Appendix S6): Australasian bittern (*Botaurus poiciloptilus*), painted honeyeater (*Grantiella picta*), superb parrot (*Polytelis swainsonii*), growling grass frog (*Litoria raniformis*), koala (*Phascolarctos cinereus*), rigid spider‐orchid (*Caladenia tensa*), winged pepper‐cress (*Lepidium monoplocoides*), river swamp wallaby‐grass (*Amphibromus fluitans*), river red gum (*Eucalyptus camaldulensis*), and black box (*Eucalyptus largiflorens*).

To estimate the potential (original) spatial distribution for each focal species (potential extent of occurrence [Brooks et al., 2019]), the may‐occur distributions from the Species of National Environmental Significance database (DAWE, 2020) were used for the 8 focal species that are nationally listed as threatened. For the other 2 species (river red gum and black box), potential spatial distributions were modeled based on spatial environmental variables (Appendix S5). Within the potential extent of occurrence of each species, we identified habitat areas based on remotely sensed land‐cover data across Australia at 25‐m resolution for 2010 and 2015 (GA, 2020). Each species was allocated land‐cover attributes representative of their broad habitat preferences (Appendix S6), enabling mapping of likely areas of habitat for each species within their potential extent of occurrence in each assessment year (2010 and 2015).

## RESULTS

**FIGURE 3:**
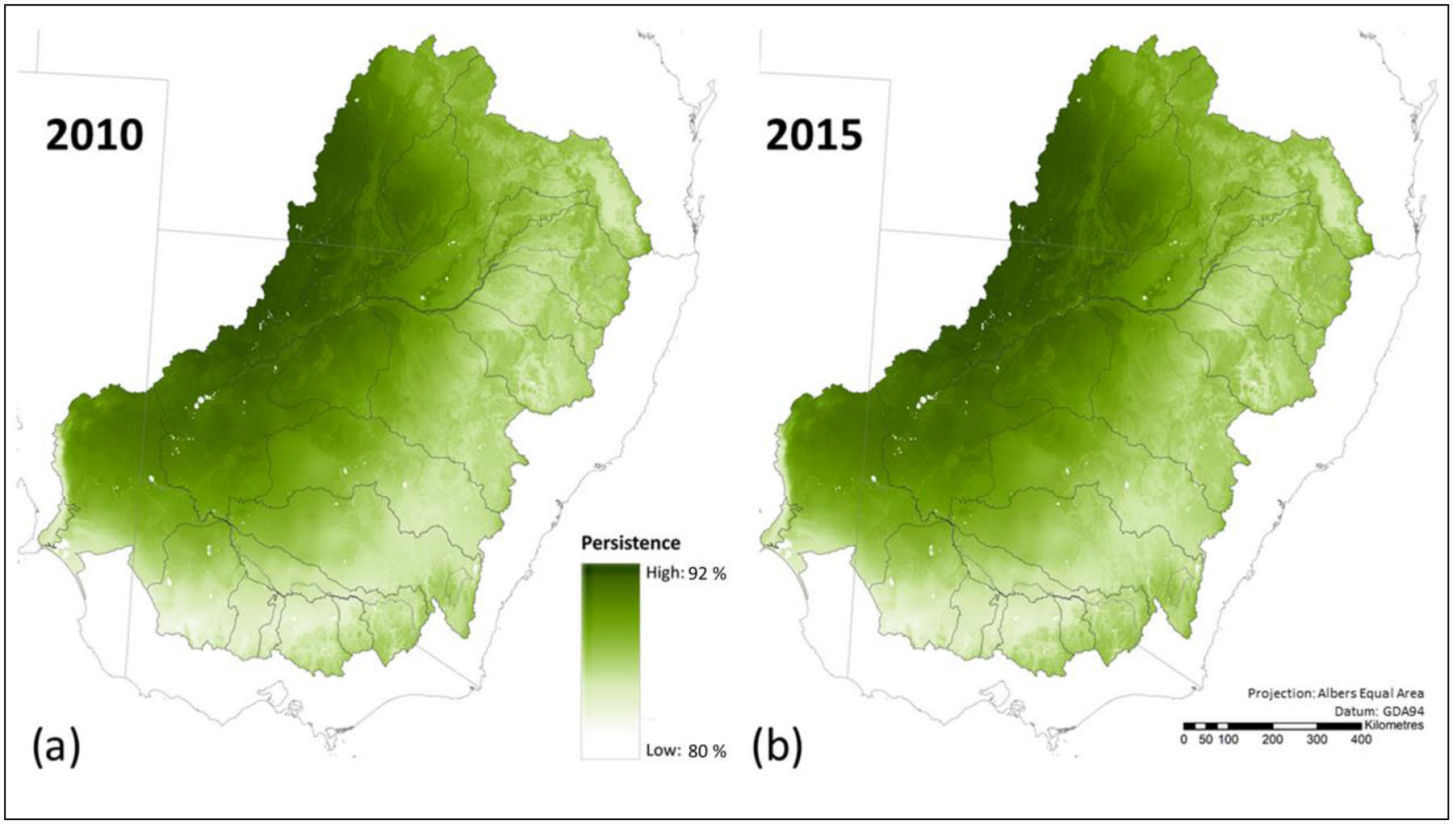
Expected persistence of vascular plant species in each location across the Murray–Darling Basin in (a) 2010 and (b) 2015. Persistence is the percentage of species originally occurring in each location expected to persist over the long term anywhere in their range, given changes in habitat condition across all of southeastern Australia (i.e., *p_i_* from equation 1 in Appendix S1). The value for *p_i_* in each grid cell is used to calculate the percentage of species retained across an ecosystem accounting area (i.e., *P* in equation 2 in Appendix S1)

Approximately 87% of the plant species originally occurring in the Murray–Darling Basin were estimated to persist in the long term anywhere across their ranges in the analysis area (Figure 3 & Table 1), given changes in habitat condition over southeastern Australia (Appendix S2). A higher percentage of plant species inhabiting the western regions were predicted to persist than for the southern and eastern regions, where habitat loss and degradation have been greater (Figure 3; Appendices S2 & S7). From 2010 to 2015, we estimated some slight improvements in expected persistence of plant species across the Murray–Darling Basin (from 86.8% to 87.1%) and in some Murray–Darling Basin regions (Figure 2 & Appendix S7).

**TABLE 1:** Biodiversity account for selected biodiversity features in the Murray–Darling Basin, Australia, from 2010 to 2015

Biodiversity feature	Vascular plants	Waterbirds	Austral‐Asian bittern	Painted honey‐eater	Superb parrot	Growling grass frog	Koala	Rigid spider‐orchid	Winged pepper‐cress	River swamp wallaby‐grass	River red gum	Black box
Units of measure	Expected species persistence (%)	Mean number of species expected	Area of habitat (`000 ha)	Area of habitat (`000 ha)	Area of habitat (`000 ha)	Area of habitat (`000 ha)	Area of habitat (`000 ha)	Area of habitat (`000 ha)	Area of habitat (`000 ha)	Area of habitat (`000 ha)	Area of habitat (`000 ha)	Area of habitat (`000 ha)
Opening measure (2010)	86.8	12.56	90.7	15,911.0	4835.0	46.0	16,443.8	858.1	605.7	9.9	3832.1	1623.4
Additions			0.4	4246.9	941.0	0.4	3183.2	503.1	585.7	0	932.1	1159.3
Reductions			0.1	3372.6	744.4	0.1	3120.3	190.3	210.6	0	1067.0	816.5
Net change	+0.3	−0.24	+0.3	+874.3	+196.6	+0.3	+62.9	+312.8	+375.1	0	−134.9	+342.8
Closing measure (2015)	87.1	12.32	91.0	16,785.3	5031.6	46.3	16,506.7	1170.9	980.7	9.9	3697.2	1966.2

**FIGURE 4:**
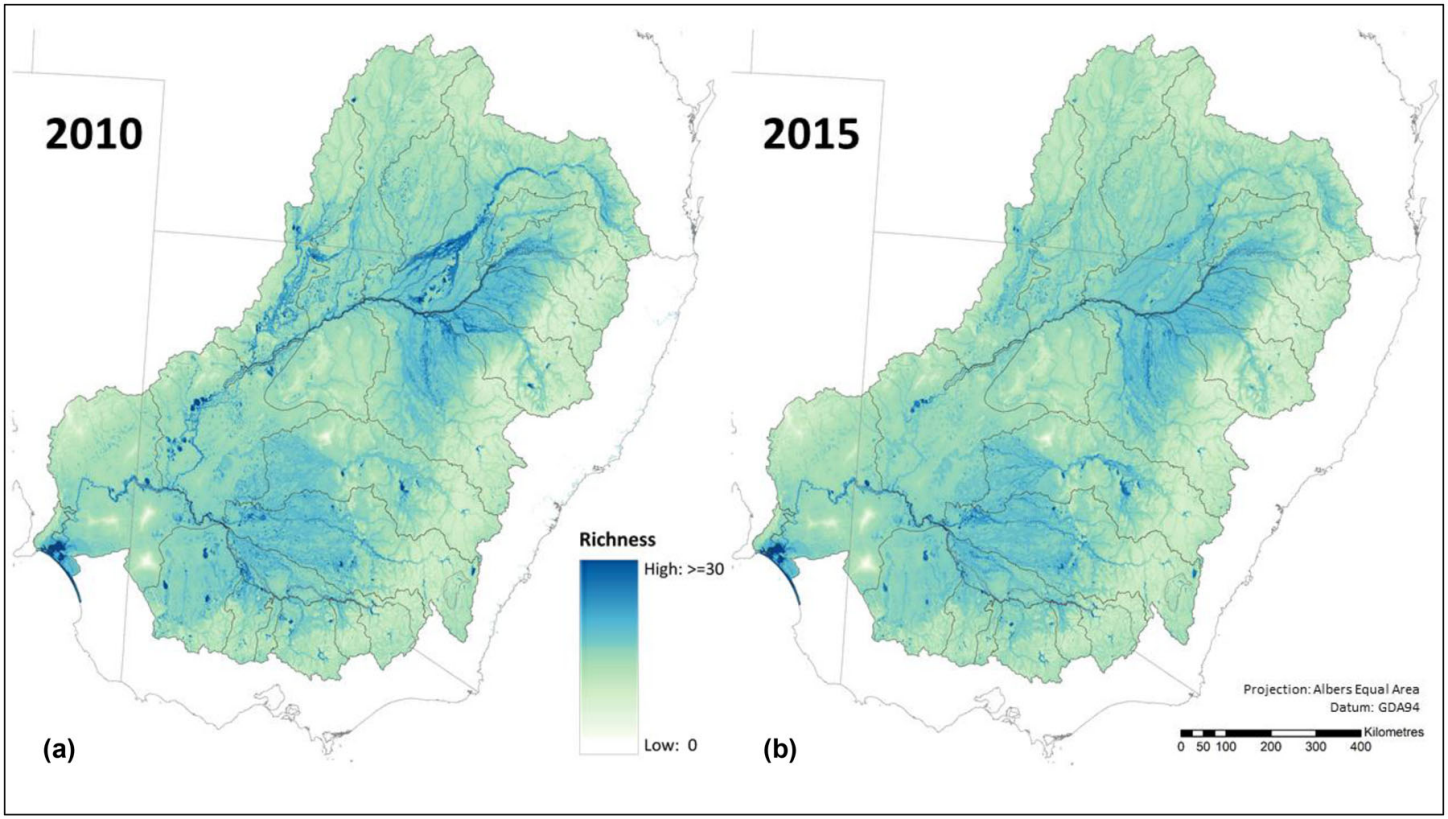
Expected waterbird species richness in each location (≈90 m grid cell) across the Murray–Darling Basin for (a) 2010 and (b) 2015

For waterbird species assemblages, estimated average species richness was almost 2% lower in 2015 than in 2010 for the Murray–Darling Basin as a whole (Table 1), with reductions across almost all the regions (Figure 4 & Appendix S7). The largest reductions in expected local waterbird species richness were evident in the northern regions of the Murray–Darling Basin (Figures 2 & 4), such as the Paroo (−6.0%) and the Condamine‐Balonne (−5.1%) (Appendix S7).

**FIGURE 5:**
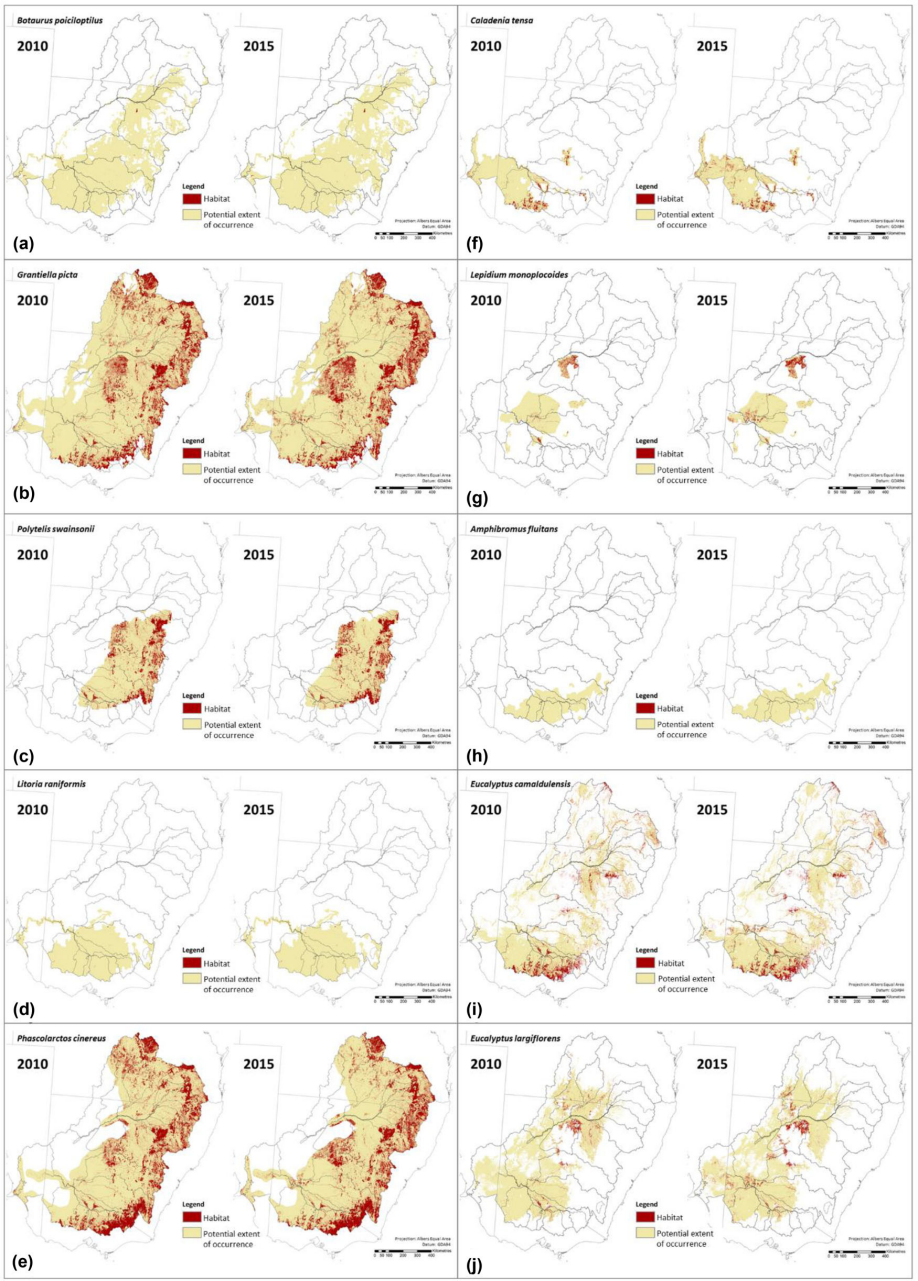
Potential extent of occurrence (cream) and habitat (red) in 2010 and 2015 for the 10 focal species: (a) Australasian bittern, (b) painted honeyeater, (c) superb parrot, (d) growling grass frog, (e) koala, (f) rigid spider‐orchid, (g) winged pepper‐cress, (h) river swamp wallaby‐grass, (i) river red gum, and (j) black box

The focal species assessment indicated substantial differences between species and ecosystem accounting areas in terms of changes in habitat area from 2010 to 2015 (Figures 2 & 5; Table 1; Appendix S8). Across the entire Murray–Darling Basin, habitat area increased for the Australasian bittern, painted honeyeater, superb parrot, growling grass frog, koala, rigid spider‐orchid, winged pepper‐cress, and black box (Table 1 & Figure 5). In contrast, the estimated area of habitat across the entire Murray–Darling Basin decreased from 2010 to 2015 for the river red gum and did not change for river swamp wallaby‐grass (Table 1). Across the Murray–Darling Basin regions, no species had consistent increases or decreases in habitat area (Figure 5 & Appendix S8).

## DISCUSSION

### Accounting for biodiversity

Our results demonstrated the utility of habitat‐based approaches in analyzing and reporting changes in biodiversity over space and time and in aligning with guidance on thematic accounting for biodiversity provided in the SEEA EA framework (UNCEEA, 2021). Fine‐resolution spatially explicit habitat‐based approaches, such as these, are particularly useful in enabling consistent reporting across multiple ecosystem accounting areas, including jurisdictions recognized by different organizations and agencies (King et al., 2021). The specific biodiversity metrics we applied are well established for thematic accounting for biodiversity under the SEEA EA (UNEP‐WCMC, 2016), are ecologically meaningful, and align with the data available for each aspect of biodiversity considered. The accounts produced with these habitat‐based approaches were also highly amenable to informing other monitoring needs in a manner consistent with the SEEA EA framework, such as reporting under the Convention on Biological Diversity Post‐2020 Global Biodiversity Framework (CBD, 2021; UNCEEA, 2021).

We focused on thematic accounting for biodiversity in the Murray–Darling Basin and did not develop a full ecosystem account, which would provide information on ecosystem extent and condition and ecosystem services. This approach of developing independent thematic accounts fits within the SEEA EA guidance (UNCEEA, 2021), although additional information from more comprehensive ecosystem accounts for the Murray–Darling Basin would provide more scope to interpret our results and to integrate them with other environmental and socioeconomic data.

Broader ecosystem accounts would help more clearly identify the possible causes and consequences of the status and change in biodiversity that we quantified. This includes linking biodiversity status and change to alterations in the extent and condition of different ecosystem types, as well as subsequent flows in ecosystem services. Development of integrated ecosystem accounts for the Murray–Darling Basin could also consider a range of socioeconomic information (Goesch et al., 2020) and support socioeconomic planning of land and water use (CSIRO, 2012; MDBA, 2020), including identifying areas where trade‐offs between biodiversity conservation and economic activities could be more effectively managed (Keith et al., 2017). There is also the potential to harness SEEA EA accounts as a baseline for scenario analyses (Mokany et al., 2019) to explore the possible implications of alternative future policy, planning, and management on biodiversity, ecosystems, and society. Integrated planning informed by ecosystem accounts (including biodiversity) could help deliver on goals for sustainable development and provide better outcomes for biodiversity.

### Whole‐basin biodiversity outcomes

For the Murray–Darling Basin as a whole, our results showed generally positive changes in biodiversity from 2010 to 2015 for the focal species and vascular plant species persistence and negative change over that period for waterbirds (Table 1 & Figure 2). These differences in basin‐wide outcomes are likely to have resulted from a number of factors, including inherent differences in key environmental drivers of habitat between taxa and the data sources used for informing habitat quality.

For all vascular plants, our estimates of long‐term species persistence for each accounting year are a function of both the compositional patterns across the region and predicted habitat condition in 2010 and 2015 (Appendix S2). The habitat condition data used for vascular plants incorporated remote sensing information over the decade prior to the assessment date to account for natural climate fluctuations (Harwood et al., 2021; Williams et al., 2021). The slight increase in expected plant species persistence from 2010 to 2015 may therefore be related to slightly wetter conditions across the Murray–Darling Basin in the 10 years preceding 2015. These wetter conditions may have effectively reduced pressures, such as extensive grazing of native vegetation, because wetter conditions result in lower stocking density per unit plant biomass (Hacker & Smith, 2007).

The estimated reductions in average waterbird species richness align with an independent assessment of decreasing trends in waterbird populations over this period (Clemens et al., 2019). The waterbird assessment relied only on the coverage of surface water in each assessment year, which declined over time because precipitation across the Murray–Darling Basin in 2010 was much greater than in 2015 (BOM, 2020). The differences in the temporal trend for waterbirds compared with vascular plants were likely due to both methodological differences in how these community‐level assessments were undertaken and key differences in the diversity patterns for these 2 groups of species. Because the vascular plant assessment applied estimates of habitat condition derived from the preceding 10 years of remote sensing data, the biodiversity assessment reflected these longer term differences. In contrast, the waterbird community‐level assessment was based on WOfS data only for the accounting years (2010 and 2015) and hence was more sensitive to annual changes in water availability. Additionally, the waterbird community‐level assessment relied far more heavily on small areas of habitat with high value for maintaining waterbird diversity, whereas the assessment for vascular plants considered change in habitat condition more comprehensively across the ecosystem accounting areas.

For the 10 focal species, the estimated area of habitat across the Murray–Darling Basin increased for 8 species and decreased for 1 species (river red gum) from 2010 to 2015 (Table 1 & Figure 5). The amount of increase in habitat varied dramatically across those 8 species, from +300 to +874,280 ha (Table 1) or from +0.3% to +61.9% of their 2010 area of habitat. Given the habitat of each species is defined by unique combinations of land cover features, it is difficult to generalize in terms of the drivers of the change in species‐level habitat availability. However, the increase in area of habitat for 8 of the focal species may have been because habitat for many of these species is related to tree cover and stricter vegetation‐clearing regulations were applied in the basin states from over the 2010 to 2015 period resulting in historically low levels of deforestation (Evans, 2016).

Importantly, these species‐level assessments were intended to identify habitat areas within the potential extent of occurrence of each species. They did not predict where each species actually occurs. Errors in land‐cover classification, or in translating land‐cover categories to habitat, may result in under‐ or overestimation of habitat area within the potential extent of occurrence.

### Regional biodiversity outcomes

Our assessment indicated that the regions in the north of the Murray–Darling Basin generally had more negative changes in biodiversity from 2010 to 2015, whereas the southern regions tended to have more positive changes (Figure 2). The regions with the largest relative reduction in biodiversity were the Paroo for waterbirds (−6%) (Table 1) and the Loddon for the focal species (7 species losing habitat) (Figure 5 & Appendix S8). No regions were estimated to have reductions in plant species persistence from 2010 to 2015. Reductions in biodiversity for the northern regions are likely related to the much wetter conditions in 2010 than in 2015 (Clemens et al., 2019), with these effects exaggerated by the semiarid climate in northwestern Murray–Darling Basin.

The regions with the largest relative increase in biodiversity were the Campaspe and Goulburn‐Broken for plant species persistence (+0.6%) (Appendix S7), the Lachlan for waterbirds (+1.3%) (Appendix S7), and the Lachlan and Murrumbidgee for the focal species (7 species gaining habitat) (Figure 5 & Appendix S8). It is possible that these changes in biodiversity were partially due to the greater amount of environmental watering actions in the southern Murray–Darling Basin (Chen et al., 2021), though more direct attribution to management actions would require comprehensive integrated ecosystem accounts across the entire Murray–Darling Basin, plus additional data and analyses.

### Future improvements in accounting for biodiversity

Our habitat‐based approaches to accounting for biodiversity in the Murray–Darling Basin provided highly resolved information on changes in biodiversity expected to have occurred across the region as a function of observed changes in habitat condition, aligned with the SEEA EA framework (UNCEEA, 2021). However, there is scope to further refine these methods to develop accounts at national and subnational scales.

Advances in habitat condition modeling and prediction will be of substantial benefit, including development of new fine‐resolution annual spatial layers. This will enable consideration and quantification of more immediate changes in habitat and biodiversity within a yearly accounting period, rather than relying on data over a longer preceding period as applied here (decadal). Such advances require novel solutions to account for the effects of natural interannual climate variability on vegetation as quantified by space‐borne sensors (Harwood et al., 2016).

For waterbirds, establishing a firmer baseline for the expected diversity under predevelopment water flow conditions (i.e., without water diversions) would enable a more direct quantification of the relative status for accounts. Essentially, this would involve a counterfactual scenario of expected waterbird diversity in a pre‐intensification reference state (McNellie et al., 2020) given the climate conditions of the accounting year. For the Murray–Darling Basin, this information is available for some areas and years (called a “without development” hydrological scenario) (MDBA, 2011), but not for the entire basin or for both the years we considered.

For the focal species accounts, more finely resolved spatial data on the potential extent of occurrence would also provide an improved baseline and enable reporting of habitat area relative to pre‐intensification reference state. The spatial data we used for potential extent of occurrence (DAWE, 2020) were of coarse resolution (1–5 km) and not resolved finely enough to delineate habitat for each species in a pre‐intensification reference state. One avenue for achieving this could be through prediction of pre‐intensification land‐cover attributes across the region of interest (Chang‐Martínez et al., 2015).

### Summary

Accounting for biodiversity has significant potential in helping track biodiversity change in a consistent way over space and time. Incorporating these accounts with broader SEEA EA ecosystem accounts offers the opportunity for better understanding the drivers and consequences of biodiversity change, including environmental, social, and economic factors (UNEP‐WCMC, 2016; Vardon et al., 2019). The quantitative approaches available for generating finely resolved biodiversity accounts over large regions will continue to improve. We demonstrated the potential for habitat‐based biodiversity assessment to play an important role in deriving finely resolved accounts across a large and diverse region. These accounts provide valuable information that has substantial potential to support more sustainable natural resource policy, planning, and management decisions that will help balance socioeconomic activities with conservation and deliver improved biodiversity outcomes in the Murray–Darling Basin.

## Supporting information

Additional supporting information may be found in the online version of the article at the publisher's website.
