# Supplementary material for: Habitat‐based biodiversity assessment for ecosystem accounting in the Murray–Darling Basin
Source: Conserv Biol. 2022 May 27;36(5):e13915. doi: 10.1111/cobi.13915 (PMC9796243; doi:10.1111/cobi.13915)
Supplement: Supplementary file 1 — Additional supporting information may be found in the online version of the article at the publisher's website. [file COBI-36-0-s001.docx]

Supporting Information

**Habitat-based biodiversity assessment for ecosystem accounting in the Murray-Darling Basin**

Appendix S1 - Community-level assessment of species persistence

The proportion of species expected to persist indefinitely into the future given loss of habitat can be derived based on community-level biodiversity patterns. This approach uses the predicted pre-European spatial patterns in both species richness and pairwise compositional dissimilarity to estimate the total proportion of species likely to persist over a region at a time point (*P*) (Fig. S1.1). Specifically, for each and every ≈90 m grid cell *i* across the Murray-Darling Basin, we estimate the proportion (*p_i_*) of species historically occurring in this cell (pre-European) that are likely to persist within remaining habitat anywhere in their range:

Eqn 1

$$p_{i}=\left[ {\sum_{j=1}^{n} s_{ij}h_{j}}/{\sum_{j=1}^{n} s_{ij}} \right]^{z}$$

where *s_ij_* is the predicted compositional similarity between the focal cell *i* and each *j* grid cell in the region of *n* grid cells, *h_j_* is the condition of habitat in each grid cell *j* (ranging continuously from pristine (= 1) to completely degraded habitat (= 0)), and *z* is the exponent of the species–area relationship. For grid cell *i*, Σ*s_ij_* quantifies the amount of similar habitat across the region if all grid cells were in pristine condition, while Σ*s_ij_h_j_* quantifies the amount of similar habitat across the region, accounting for habitat loss and degradation in some grid cells (through *h_j_*). In terms of condition, the term ‘pristine’ equates to a reference state. For vascular plants, a *z*-value of 0.25 was applied, which approximates values commonly observed for terrestrial taxa (Rosenzweig 1995).

The overall proportion of species (*P*) expected to persist across a region can then be estimated as a weighted average of the *p_i_* values for all *n* individual grid cells, to incorporate the effects of compositional overlap between grid cells and the species richness of each cell (*r_i_*):

$$P={\sum_{i=1}^{n} p_{i}w_{i}}/{\sum_{i=1}^{n} w_{i}}$$

where the weight (*w_i_*) of a grid cell is calculated as:

Eqn 2

$$w_{i}=\frac{r_{i}}{\sum_{j=1}^{n} s_{ij}}$$

This approach (Fig. S1.1) was applied to estimate the proportion of the original vascular plant species expected to persist across different ecosystem accounting areas for the 2 years considered (2010 and 2015). For all analyses in the present study, we applied estimates of habitat condition from the HCAS continental products (Harwood *et al.* 2021; Williams *et al.* 2021) (Appendix S2), clipped to the NARCLiM domain (Evans *et al.* 2014), resampled to ≈90 m resolution. Given the computational challenges in undertaking this analysis for the whole Murray-Darling Basin at ≈90 m resolution (approximately 360 million grid cells), a stratified random sample of grid cells *j* was used to determine the compositional similarity to each and every focal cell *i*.

Eqn 3


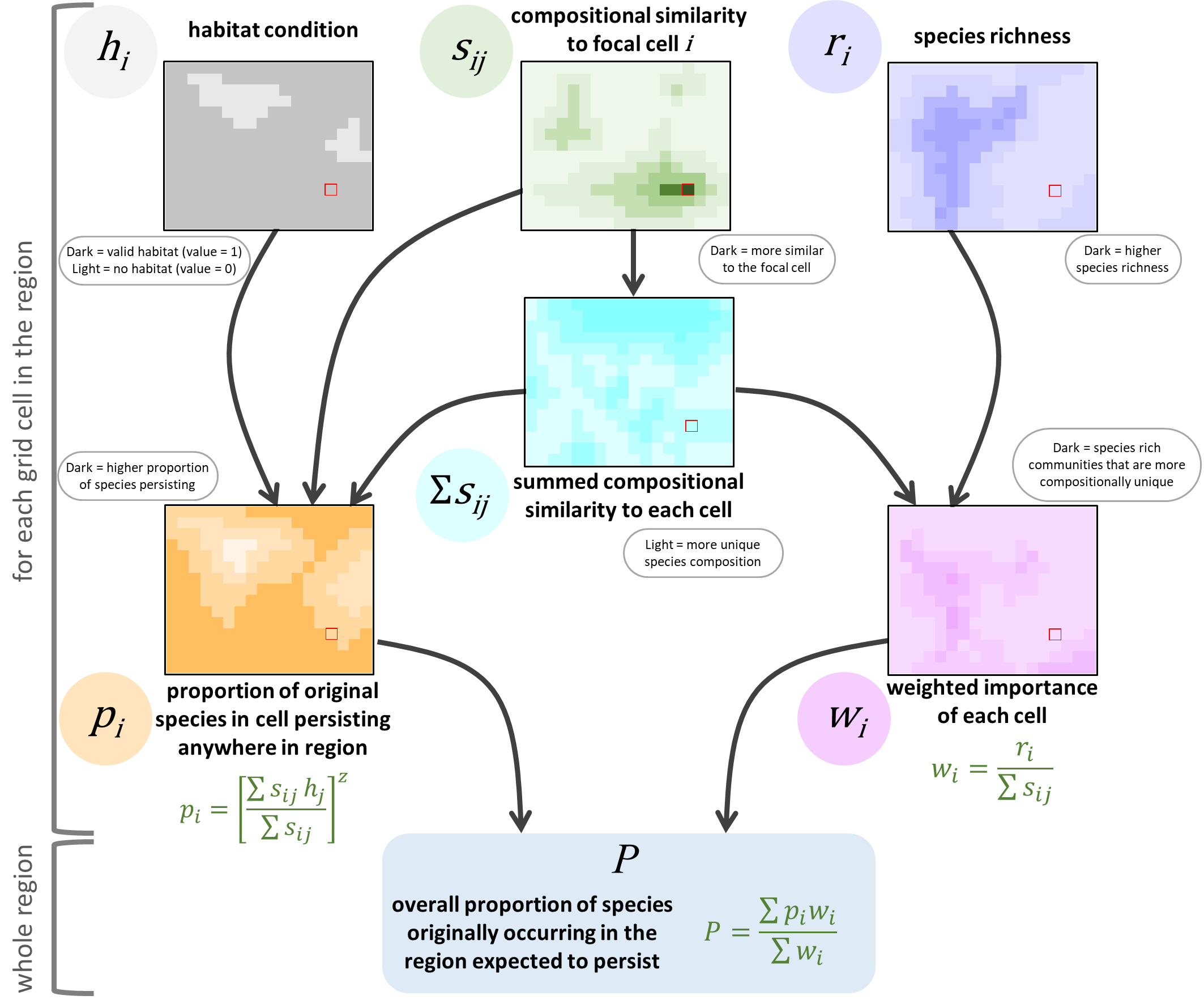


**Figure S1.1.** Conceptual illustration of the analytical approach to estimating the impact of changes in habitat condition on the proportion of species originally occurring in a region expected to persist (*Pγ:* Ferrier et al. (2004)). The three key inputs are spatial layers of habitat condition (0–1 range, where 1 = pristine), species richness and predicted compositional similarity between any pair of grid cells, applied here using transformed layers from generalised dissimilarity modelling. The *s_ij_* spatial layer shows the predicted compositional similarity of every grid cell to the focal grid cell *i*, in red outline. A different focal grid cell will have a different spatial layer for *s_ij_*, showing how similar the surrounding grid cells are in composition. In contrast, there is only one spatial layer for the summed compositional similarity Σ*s_ij_*. The value of the grid cell *i* in red outline in the Σ*s_ij_* spatial layer is effectively the sum of all the grid cells in the *s_ij_* spatial layer. These inputs enable calculation for each grid cell of the proportion of species originally occurring there that are expected to persist anywhere in the region (*p_i_*) and a weighting for each cell of its importance for biodiversity (*w_i_*). These enable calculation of a single value for the region of the overall proportion of species expected to persist (*Pγ*).
Figure adapted from Mokany et al. (2019).

**Appendix S2 – Habitat Condition for vascular plants**


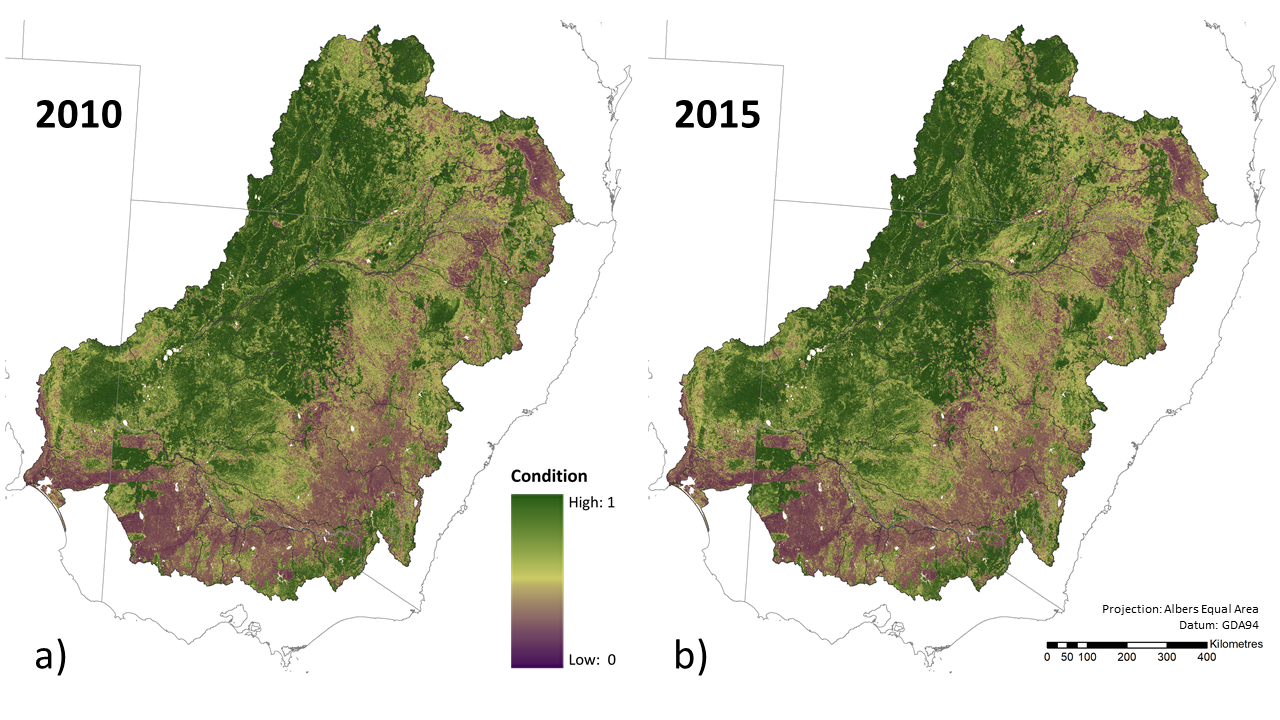


**Figure S2.1.** Estimated habitat condition for biodiversity in each location (≈90 m grid cell) across the Murray-Darling Basin for a) 2010 and b) 2015, using HCAS version 2.1. This is an input to the biodiversity assessment for vascular plants (Appendix S1). Derivation of the habitat condition spatial layers is detailed in Harwood et al. (2021) and (Williams *et al.* 2021).

**Appendix S3 – Diversity models for vascular plants**

Data inputs to plant community modelling

#### Biological data

Models of plant community diversity were developed over the NARCliM spatial domain (Evans *et al.* 2014) (Figure 15). Plant community survey plot data over this area were obtained from four sources: (i) the NSW BioNet database (accessed 9/10/2019), (ii) the Terrestrial Ecosystem Research Network’s (TERN) AEKOS data portal (accessed 17/1/2018), (iii) the Victorian Biodiversity Atlas (accessed 27/6/2017) and (iv) the Queensland CORVEG database (accessed 16/8/2018). Taxonomic nomenclature of the recorded species within all plots was standardised using the Australian Plant Census (APC) list of unique species names. The Atlas of Living Australia was used to rationalise synonyms to accepted species names, using the ALA4R package (Raymond *et al.* 2017) in the R statistical computing environment (R Development Core Team 2020). Records of plant species not native to Australia were flagged in the dataset.

A small number of plots with very large survey areas (larger than the ~8,000 m^2^ grid cell area) were removed from the dataset. The proportion of species in a plot that were native to Australia was quantified; plots where the composition was <85% native species were removed. This processing and filtering resulted in a total of 66,608 plant community plots within the NARCliM spatial domain (Fig. S3.1). Only data for native species were retained for the subsequent modelling.

The community compositional dissimilarity index applied here (Sørensen 1948) was derived from the plot data following transformation to properly represent the spatial grid cell resolution being applied (~90 m). The biodiversity scaling approach of Mokany et al. (2013) was applied, which uses the species–area power model (*S = cA^z^*) to scale compositional dissimilarity from small sample areas of survey plots to larger grid cell areas. Under this approach, the species richness of a grid cell (*S*) is predicted from the observed richness of the community survey (*c*) and the area of the grid cell relative to the survey area (*A*). To scale pair-wise compositional dissimilarity from the community surveys to the grid cells they occur within, we first predicted the number of species in common between the two grid cells *i* and *j* (*S_com,ij_*) from the observed number of species in common between the two community surveys (*c_com,ij_*) using the species–area power relationship (*S_com,ij_* = *c_com,ij_A^zcom^*). We then calculated the predicted Sørensen’s compositional dissimilarity between the two grid cells (*b_ij_* = 1– [*2S_com,ij_*/(*S_i_*+*S_j_*)]) using the predicted species richness of each grid cell (*S_i_*, *S_j_*) and the predicted number of species in common between the two grid cells (*S_com,ij_*). We applied a single scaling factor for species richness (*z* = 0.25) as commonly observed across a variety of ecological analyses (Rosenzweig 1995), and for the number of species shared between a pair of communities (*z_com_ =* 0.42), as derived previously (Mokany *et al.* 2013).

This scaling approach retains the underlying gradients in species richness and compositional dissimilarity that are observed across the community survey plots, but scales the absolute values so that they better represent those of the entire grid cells being modelled and projected.


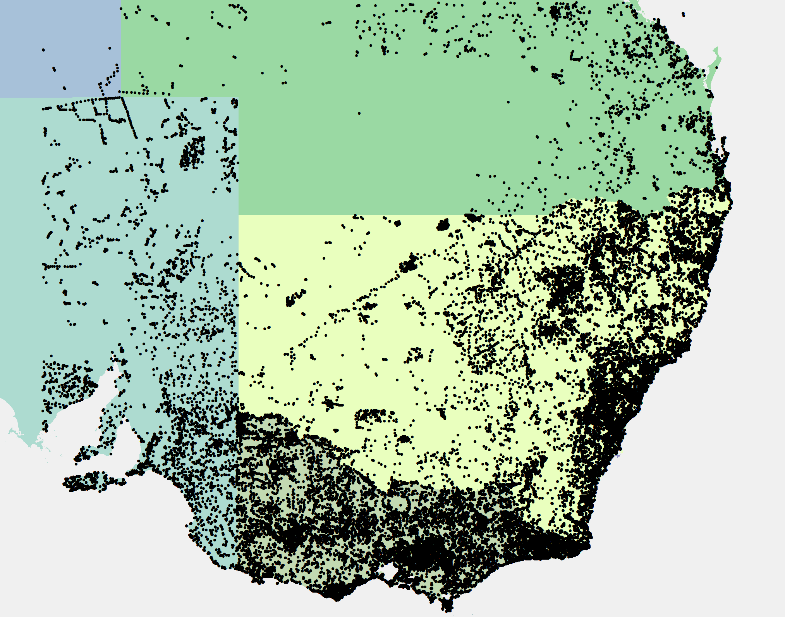


**Figure S3.1.** Spatial distribution of the plant community plot data used for modelling compositional dissimilarity and species richness. Australian states are shown in different colours.

#### Environmental data

Spatially complete and consistent environmental data for the NARCLiM domain were collated, derived and prepared (Harwood *et al.* 2018), being aligned to the SRTM 3 second (~90 m) digital elevation model of Australia (Gallant *et al.* 2011). Monthly climate data were derived using ANUClim V6.1 (Xu & Hutchinson 2010), with maximum temperature and evaporation adjusted for slope and aspect using CSIRO’s Terraforma package (developed by Harwood et al. in Reside et al. (2013)) to derive the suite of climatic indices (using Essential Climate Variables) for biodiversity modelling described by Williams et al. (2012). Several soil and landform layers at 3-second resolution were obtained from the TERN Soil and Land Grid of Australia (Gallant & Austin 2015; Grundy *et al.* 2015; Viscarra Rossel *et al.* 2015) including bulk density, available water-holding capacity, organic carbon, nitrogen, phosphorus, pH and percent sand/silt/clay, all at 3 depth ranges (0–30 cm, 30–100 cm, 0–200 cm), plus an additional layer for total soil depth. These variables were supplemented with topographic descriptor variables derived from the 3-second (~90 m) digital elevation model, including topographic wetness index (Gallant & Austin 2012b) and elevation focal range within a 300 m radius (Gallant & Austin 2012a). WOfS data (Mueller *et al.* 2016) were used to derive layers representing proportion of time a location was covered by freshwater or saltwater. Environment data were extracted for the plant community plot locations, and correlations between environment variables were quantified to inform variable selection.

Modelling of plant community compositional similarity

We generated a model of pairwise community compositional dissimilarity for vascular plants using generalised dissimilarity modelling (GDM) (Ferrier *et al.* 2007), applying the *gdm* package in *R* (MacArthur 1965; Manion *et al.* 2018; R Development Core Team 2020). Given the very large number of possible site pairs for the plot data for vascular plants (>3.4 billion), we applied an integrated approach to site-pair sampling and variable selection, based on cross-validation. For each iteration of a cross-validation sample, 80% of sites were randomly selected to train the GDM, while the remaining 20% of sites were used to validate the predictions of the provisional models. For both sets of sites (training and testing) we applied the same methods to sample site pairs, with 300,000 site pairs generated for training the model (from the training data) and 50,000 site pairs generated to test the model (from the testing sites). For each combination of model predictors assessed, this random sampling procedure was repeated 10 times.

We derived a reduced set of candidate predictor variables by assessing the predictive power of each variable independently, then adding variables to the candidate set based on their individual explanatory power, ensuring no variables selected for further assessment were highly correlated (absolute Pearson’s R >0.7). From this initial candidate set of variables, we applied a backward elimination variable selection approach. The performance of preliminary models to predict compositional dissimilarities for the testing site pairs was used to remove the least informative variable from the candidate set (assessed using deviance explained), stopping when a parsimonious set of statistically significant predictor variables remained in the final model, assessed via permutation test (Mokany *et al.* 2014).

Site-pair samples were generated for each of the test and training sets of sites, based on a geographically weighted sampling scheme. This scheme is intentionally biased towards selecting site pairs that are geographically closer together, hence likely to be environmentally similar, while undertaking this sampling in an even manner across the region of interest. This approach ensures a good spatial and environmental coverage of sites in site pairs, from across the geographic and environmental space. We applied a net of sample nodes at 150 km distance, randomly sampling sites to combine into pairs using a Gaussian distribution around each node, and incorporated 10% of site pairs randomly selected over the NARCLiM domain (Mokany *et al.* 2018).

The final model of compositional dissimilarity comprised 10 predictor variables and produced a suitable balance between parsimony and explanatory power (Table S3.1, Fig. S3.2). The predictors selected covered an ecologically relevant range of variables for vascular plants, including moisture, temperature, topography and soils (Table S3.1, Fig. S3.3). The final model (Fig. S3.2, Fig. S3.4) had an intercept of 0.89, and explained 31.2% deviance in compositional dissimilarity, with a root mean square error of 0.11.

**Table S3.1.** Predictor variables included in the generalised dissimilarity modelling for vascular plants, their individual deviance explained (D^2^) under cross-validation and the loss of explained deviance from the full model following the removal of each variable.

| Variable | Individual D^2^ | Loss of D^2^ if dropped |
| --- | --- | --- |
| Mean annual precipitation | 20.54 | 3.68 |
| Precipitation seasonality 1 (solstice) | 5.06 | 0.42 |
| Precipitation seasonality 2 (equinox) | 4.43 | 0.63 |
| Maximum temperature warmest month | 14.67 | 1.70 |
| Minimum temperature coolest month | 5.56 | 0.42 |
| Elevation focal range (300 m) | 9.08 | 1.17 |
| Soil clay content (0–30 cm depth) | 4.32 | 0.21 |
| Soil cation exchange capacity (30–100 cm depth) | 5.32 | 0.18 |
| Soil sand content (0–30 cm depth) | 3.31 | 0.48 |
| Coverage by surface water (WOfS) | 1.23 | 0.50 |

All variables statistically significant at *P* <0.001.


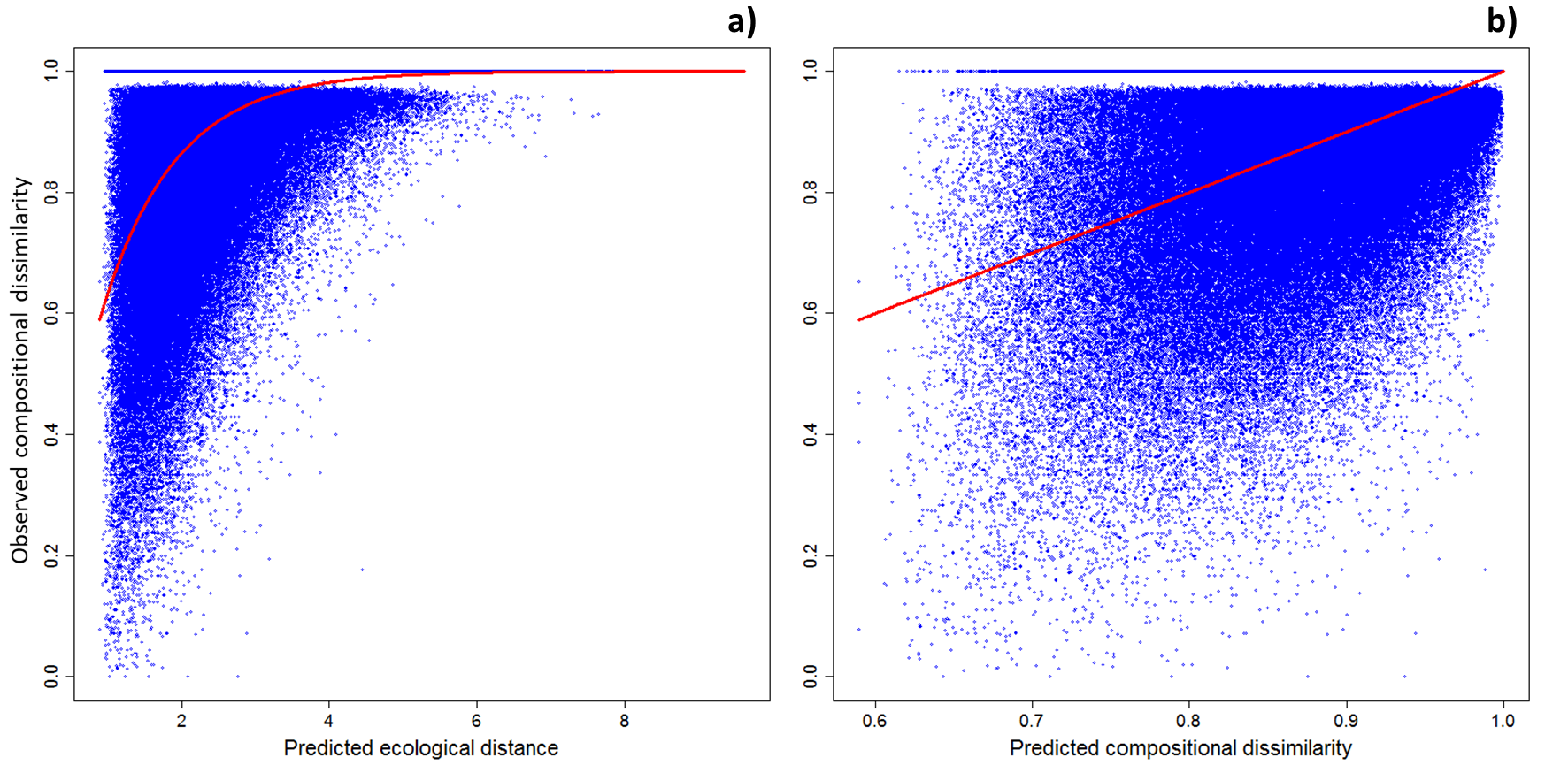


**Figure S3.2.** Fitted generalised dissimilarity model for vascular plants, being the observed compositional dissimilarity as a function of the a) predicted ecological distance and b) predicted compositional dissimilarity.


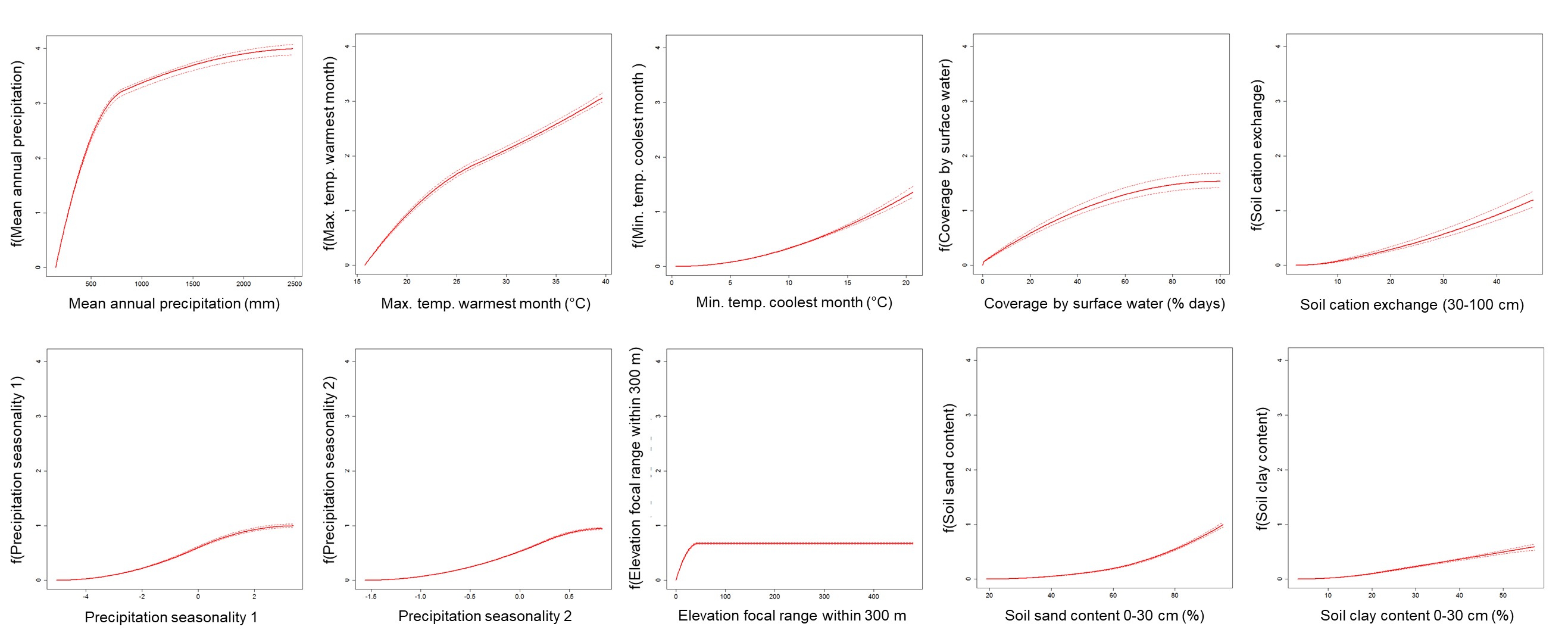


**Figure S3.3.** Fitted spline functions for each predictor variable included in the generalised dissimilarity model (GDM) for vascular plants. For each panel, the variable’s native scale is on the x-axis, and the GDM transformed values on the y-axis.


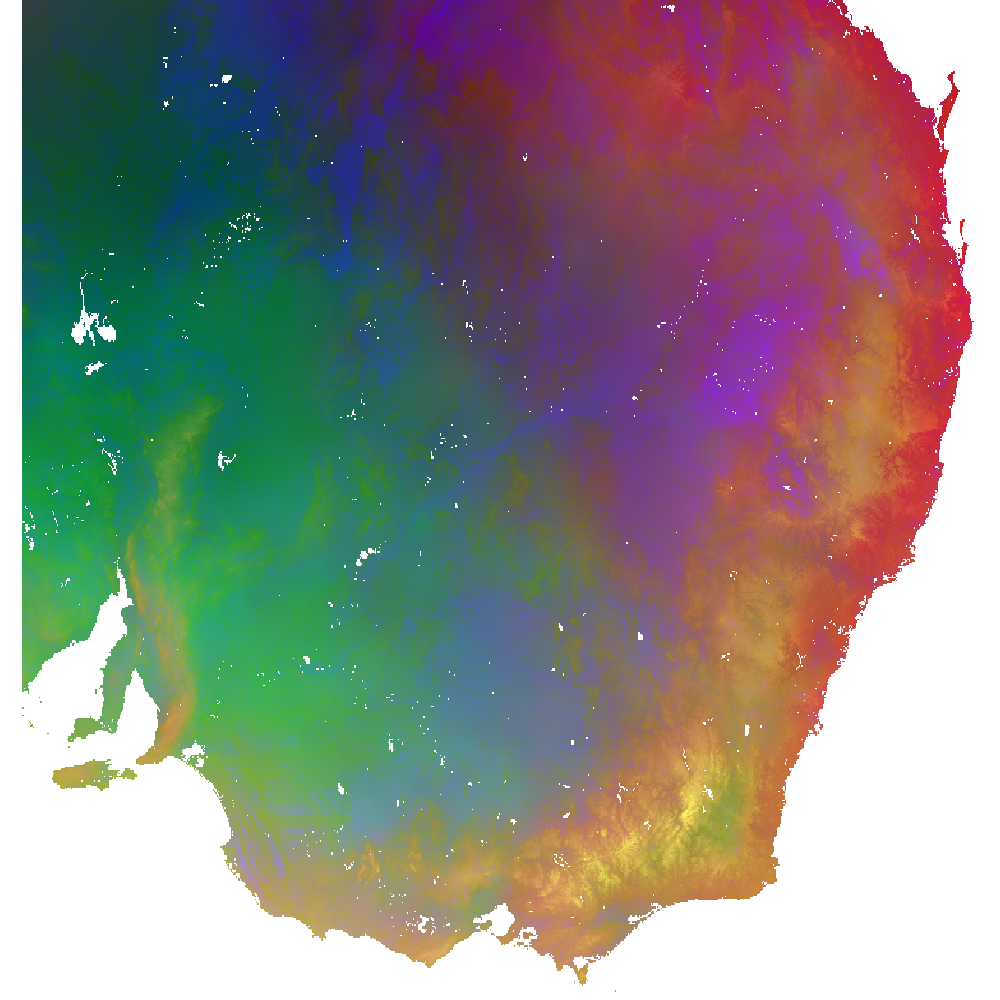


**Figure S3.4.** Simplified representation of the vascular plant generalised dissimilarity model (GDM), being the primary 3 axes from a principle components analysis undertaken on the 8 GDM transformed grids (200,000 cell sample), with each axis allocated the red, blue or green colour scale. More similar colours represent similar predicted plant species composition.

Modelling of plant community species richness

We generated a model of plant community species richness using generalised additive modelling (GAM) (Hastie & Tibshirani 1986), applying the *mgcv* package in *R* (Wood 2016; R Development Core Team 2020). Models were developed applying a cross-validation sample of 80% of randomly selected sites for model training, and the remaining 20% of sites used for validation, with this random sampling procedure repeated 10 times. We derived a reduced set of candidate predictor variables by assessing the predictive power of each variable independently, then adding variables to the candidate set based on their individual explanatory power, ensuring no variables selected for further assessment were highly correlated (absolute Pearson’s R >0.7). From this initial candidate set of variables, we applied a backward elimination variable selection approach. The performance of preliminary models to predict species richness for the testing site pairs was used to remove the least informative variable from the candidate set (assessed using deviance explained), stopping when a parsimonious set of statistically significant predictor variables remained.

The final model of plant species richness contained 10 predictor variables and explained 29.3% deviance in observed species richness (root mean square error = 25.7). The strongest predictors of species richness were mean annual precipitation and elevation focal range (Table S3.1, Fig. S3.5, Fig. S3.6). The richness model included other predictors relevant to moisture availability, including time covered by surface water and potential evaporation. Spatial projection of the plant species richness model produced expected patterns of highest predicted species richness in mountainous coastal areas, and lowest predicted richness in the semi-arid lowlands (Fig. S3.7).

**Table S3.2.** Variable contribution to the plant species richness model for south-east Australia. The second column indicates the importance of each variable in the multi-variate model, expressed through the *F*-statistic, including the significance of each predictor in that model. The third column indicates the amount of deviance explained by each predictor when used independently in a single variable model.

| Variable | All variable model  *F*-statistic | Single variable model % deviance explained |
| --- | --- | --- |
| Mean annual precipitation | 501.0 | 16.6 |
| Mean annual potential evaporation | 411.8 | 2.4 |
| Maximum temperature warmest month | 468.0 | 8.5 |
| Soil available water capacity (0–30 cm depth) | 178.1 | 1.2 |
| Soil clay content (0–200 cm depth) | 303.7 | 7.4 |
| Soil cation exchange capacity (30–100 cm depth) | 151.9 | 10.9 |
| Soil sand content (30–100 cm depth) | 116.1 | 3.6 |
| Elevation focal range (300 m) | 394.2 | 14.2 |
| Gravity anomaly | 194.0 | 1.8 |
| Coverage by surface water (WOfS) | 181.6 | 2.7 |

All variables statistically significant at *P* <0.001.
WOfS = Water Observations from Space.


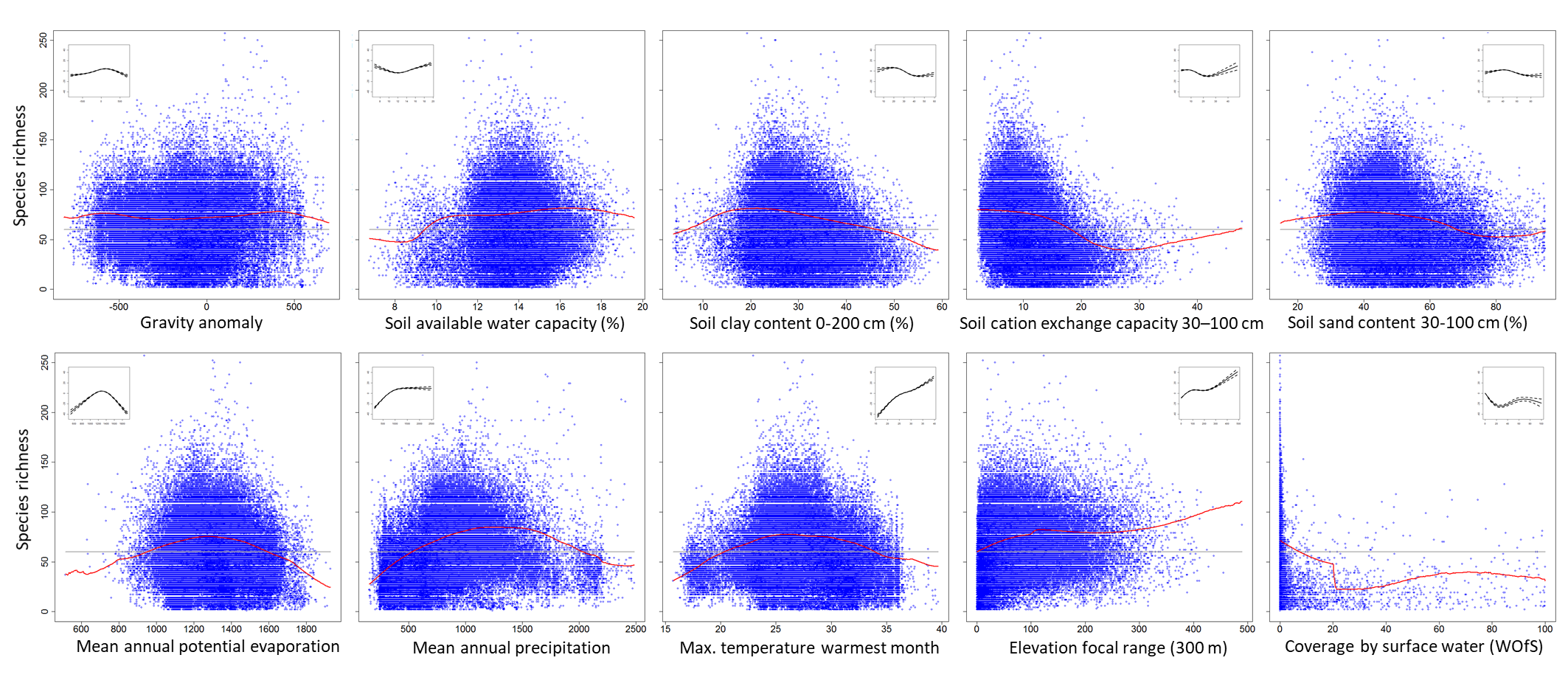


**Figure S3.5.** Plant species richness model response functions for the 10 predictor variables used in the model. Solid red lines indicate the predicted richness, when all other variables are held at the mean value. Observed values are shown in blue, with a horizontal line in grey marking the average species richness across all plots (63.9 species). The generalised additive modelling splines are shown in insets to each panel.


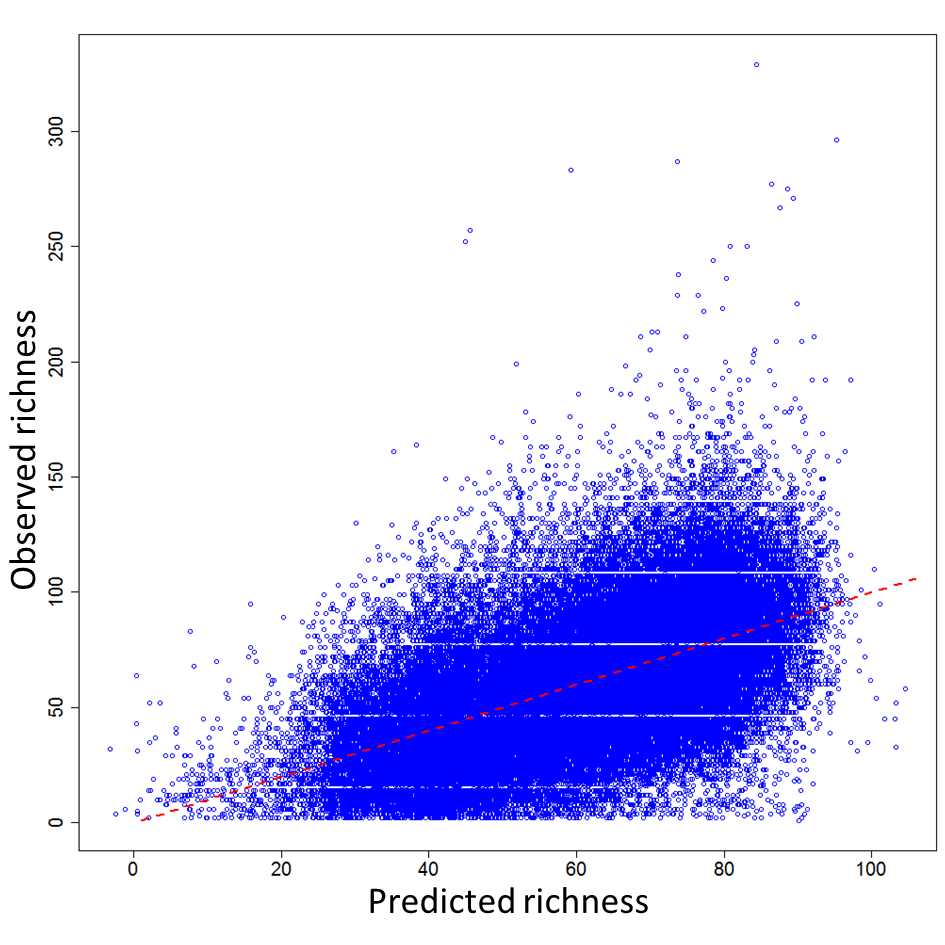


**Figure S3.6.** Observed versus model predicted plant species richness, with the 1:1 line shown in red.


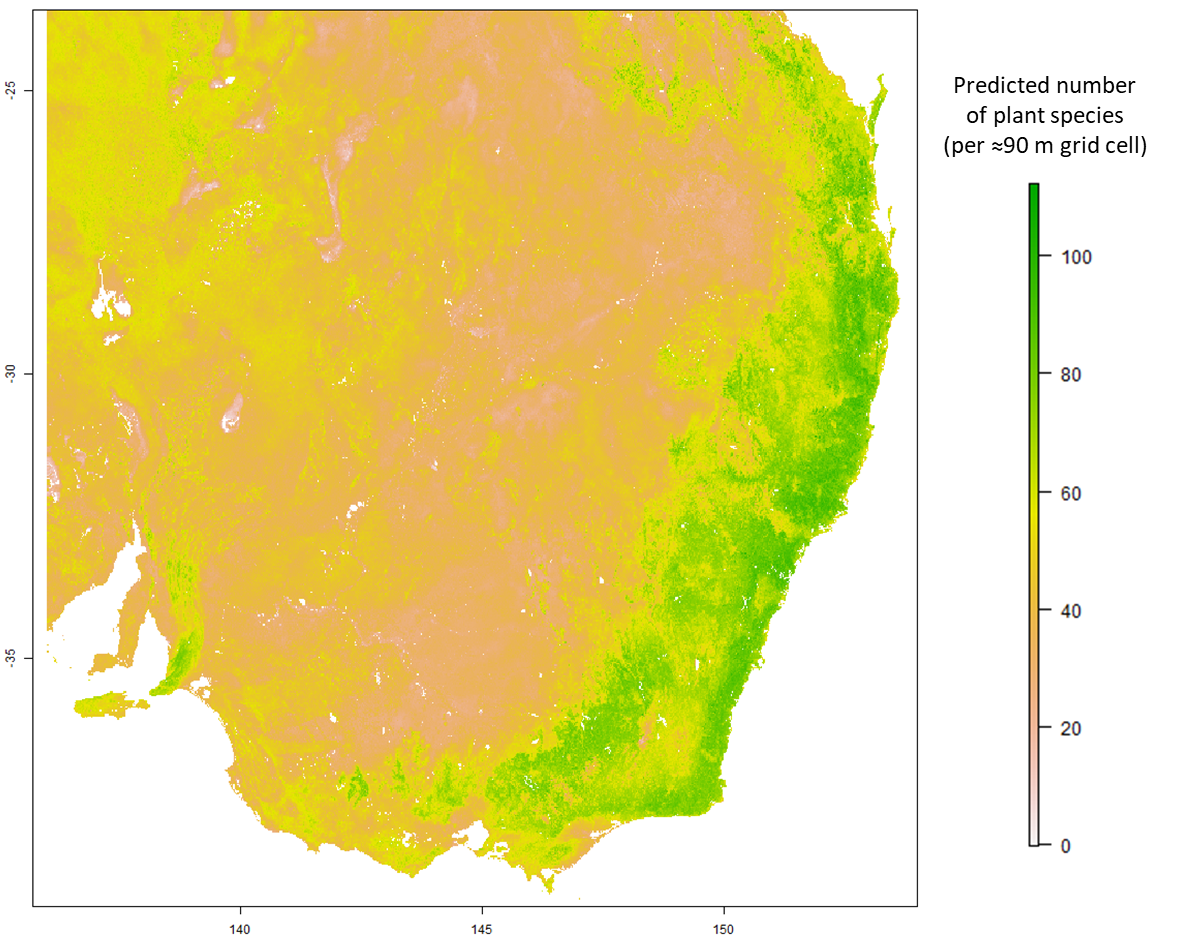


**Figure S3.7.** Spatial projection of the model of plant species richness across the NARCLiM domain.

**Appendix S4 - Species richness model for waterbirds**

Biological data

Species occurrence records for 95 waterbird species occurring in south-east Australia (Reid *et al.* 2013) were downloaded from the Atlas of Living Australia (ALA) on 29 July 2020^[[1]](#footnote-1)^. Downloaded records were filtered to the NARCLiM extent, removing records prior to 1970, with spatial uncertainty >1 km, and that failed standard ALA quality tests (i.e. location or spatial issues, possible outlier, unaddressed user assertions, created from environmental DNA analysis or are fossils, an absence rather than presence record, date of the occurrence prior to 1700 or the date given is invalid, for example in the future, issues in the name given for the organism, spatial coordinates that are likely to be inaccurate by 10 kilometres or more, a duplicate record.). To translate individual records of species occurrence to information of species assemblage composition, records were allocated to the ≈90 m grid cell within 400 m radius that had the greatest number of species recorded within a 400 m radius. Those grid cells with <3 species were removed as likely underestimates of the waterbird fauna. This resulted in 26,672 grid cells with waterbird community composition data in the NARCLiM spatial domain that were used to model species richness.

Environmental data

To assemble a set of candidate spatial predictors of waterbird species richness, we first included all the climate, topography and soil layers that were applied for modelling vascular plant diversity (Appendix S3) at ≈90 m resolution across the NARCLiM spatial domain (Harwood *et al.* 2018). In addition to these candidate predictor layers, we generated several customised spatial layers of potential utility for explaining waterbird species richness patterns. First, we created a number of spatial summary layers from the WOfS spatial layers (Mueller *et al.* 2016), including a layer indicating coverage by sea/salt water, and a layer indicating the average WOfS values within a neighbourhood of 500 m radius around each grid cell. In addition to these we created a spatial layer denoting the minimum distance of each grid cell to a permanent or semi-permanent aquatic habitat, as defined by an intersection of the Australian National Aquatic Ecosystem (ANAE) (Brooks *et al.* 2014; Brooks 2017) spatial data, and the Australian Hydrological Geospatial Fabric (geofabric) (BOM 2012), to provide coverage beyond the Murray-Darling Basin.

Species richness modelling

Given the likely underestimation of waterbird species richness in many of the locations for which we derived community composition from ALA records, we modelled the 90^th^ percentile of species richness values as a function of environmental predictors. The 90^th^ percentile is likely a better estimate of potential waterbird species richness in a location, rather than the mean value. We generated a model of waterbird species richness using quantile GAM (Hastie & Tibshirani 1986), applying the *mgcv* package in *R* (Wood 2016; R Development Core Team 2020). We derived a reduced set of candidate predictor variables by assessing the predictive power of each variable independently, then adding variables to the candidate set based on their individual explanatory power, ensuring no variables selected for further assessment were highly correlated (absolute Pearson’s R >0.7). From this initial candidate set of variables, we applied a backward elimination variable selection approach, stopping variable elimination when a parsimonious set of statistically significant predictor variables remained.

The final model of waterbird richness contained 10 predictor variables and explained 49.0% deviance in the 90^th^ percentile of species richness. The strongest predictor of waterbird species richness was our customised variable of mean WOfS within a 500 m radius (Table S4.1, Fig. S4.1). The richness model also included other predictors relevant to climate, topography, substrate and wetland habitats. As the WOfS 500 m predictor dominated the model, this enabled us to project the waterbird model and predict waterbird species richness across south-east Australia for both 2010 and 2015, using WOfS data from those years. These spatial projections produced expected patterns of highest predicted waterbird species richness in areas associated with extended coverage of water (Fig. 4).

**Table S4.1.** Variable contribution to the waterbird species richness model for south-east Australia. The second column indicates the importance of each variable in the multi-variate model, expressed through the Chi-squared statistic, including the significance of each predictor in that model. The third column indicates the amount of deviance explained by each predictor when used independently in a single variable model.

| Variable | All variable model  Chi-squared | Single variable model % deviance explained |
| --- | --- | --- |
| Coverage by surface water (WOfS 500 m radius) | 986.1 | 41.7 |
| Distance to aquatic habitat | 132.1 | 38.5 |
| Elevation focal range (300 m) | 119.1 | 38.1 |
| Annual actual evapotranspiration | 69.0 | 36.5 |
| Saltwater WOfS | 58.6 | 36.1 |
| Aridity Index (minimum monthly) | 47.7 | 36.6 |
| Elevation | 46.9 | 37.8 |
| Distance to coast | 45.8 | 36.2 |
| Soil pH (0–30 cm depth) | 45.6 | 37.0 |
| Moisture balance (precipitation - potential evaporation) | 25.7 | 36.4 |

All variables statistically significant at *P* <0.001.
WOfS = Water Observations from Space.


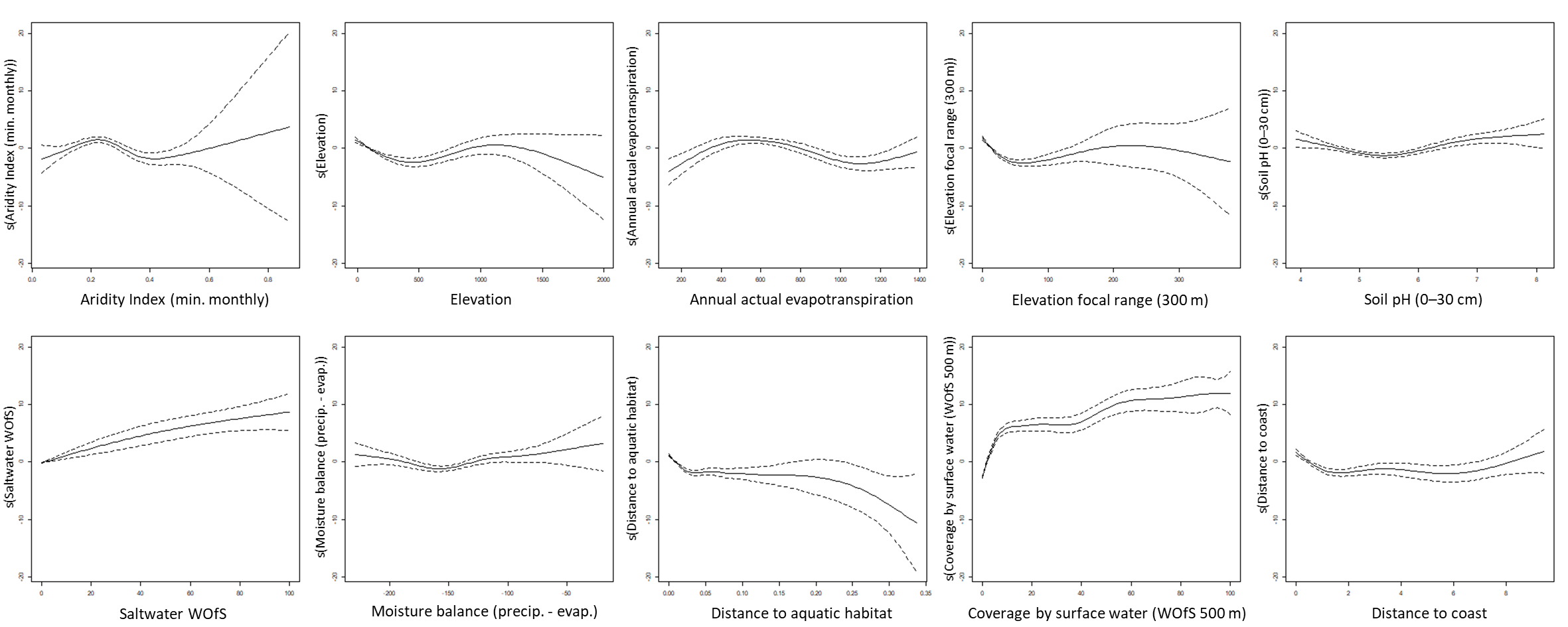


**Figure S4.1**. Model response functions for the 10 predictor variables used in the waterbird species richness model, including the 95% confidence intervals (dashed line). evap = potential evaporation; precip = precipitation; WOfS = Water Observations from Space.

**Appendix S5 - Species distribution models for river red gum and black box**

Methods

Models of the potential original distributions of river red gum (*Eucalyptus camaldulensis*) and black box (*E. largiflorens*) were developed over the NARCliM spatial domain (Evans *et al.* 2014) (Figure 15). We harnessed the same plant community survey plot data as were applied for the modelling of vascular plant diversity patterns (Appendix D). For both species modelled, we identified those survey plots where each species was present or absent. River red gum occurred in 7,286 survey plots while black box occurred in 3,796 survey plots.

We modelled the presence and absence of both species as a function of the same candidate environmental predictors used for modelling vascular plant diversity patterns (Appendix D). Species distribution models were fit using GAM (Hastie & Tibshirani 1986), applying the *mgcv* package in *R* (Wood 2016; R Development Core Team 2020). We derived a reduced set of candidate predictor variables by assessing the predictive power of each variable independently, then adding variables to the candidate set based on their individual explanatory power, ensuring no variables selected for further assessment were highly correlated (absolute Pearson’s R >0.7). From this initial candidate set of predictor variables, we applied a backward elimination variable selection approach, stopping variable elimination when a parsimonious set of statistically significant predictor variables remained.

River red gum distribution model

The final model of river red gum potential original distribution contained 17 predictor variables and explained 67.6% deviance in river red gum occurrence. The strongest predictor of river red gum occurrence was our customised variable of maximum mean WOfS within 2 km radius (Table S5.1, Fig. S5.1). The river red gum model also included other predictors relevant to climate, topography and substrate. To derive a binary spatial projection of river red gum predicted potential extent of occurrence, we applied a threshold to the continuous predicted values, with the threshold value set to include 95% of the observed occurrence observations, to maintain a relatively generous prediction of potential original distribution (Fig. S5.2).

**Table S5.1.** Variable contribution to the river red gum species distribution model for south-east Australia. The second column indicates the importance of each variable in the multi-variate model, expressed through the Chi-squared statistic, including the significance of each predictor in that model. The third column indicates the amount of deviance explained by each predictor when used independently in a single variable model.

| Variable | All variable model  Chi-squared | Single variable model % deviance explained |
| --- | --- | --- |
| Coverage by surface water (WOfS maximum mean 2 km radius) | 1151.5 | 13.7 |
| Soil bulk density (0–2 m depth) | 87.8 | 11.0 |
| Soil clay content (0–2 m depth) | 274.0 | 19.9 |
| Depth to regolith | 201.3 | 18.6 |
| Depth of soil | 214.1 | 13.8 |
| Soil effective cation exchange capacity (0–2 m depth) | 405.6 | 15.0 |
| Annual potential evaporation | 239.5 | 4.4 |
| Potassium radiometric intensity | 153.1 | 6.7 |
| Annual precipitation | 634.0 | 23.6 |
| Soil phosphorus concentration (0–2 m depth) | 396.0 | 6.1 |
| Precipitation seasonality 1 (solstice) | 689.7 | 20.2 |
| Precipitation seasonality 2 (equinox) | 439.9 | 15.0 |
| Soil silt content (0–2 m depth) | 86.9 | 6.8 |
| Soil sand content (0–2 m depth) | 101.4 | 3.9 |
| Thorium radiometric intensity | 158.1 | 8.8 |
| Minimum temperature of the coolest month | 535.3 | 18.3 |
| Maximum temperature of the warmest month | 483.8 | 11.6 |

All variables statistically significant at *P* <0.001.
WOfS = Water Observations from Space.


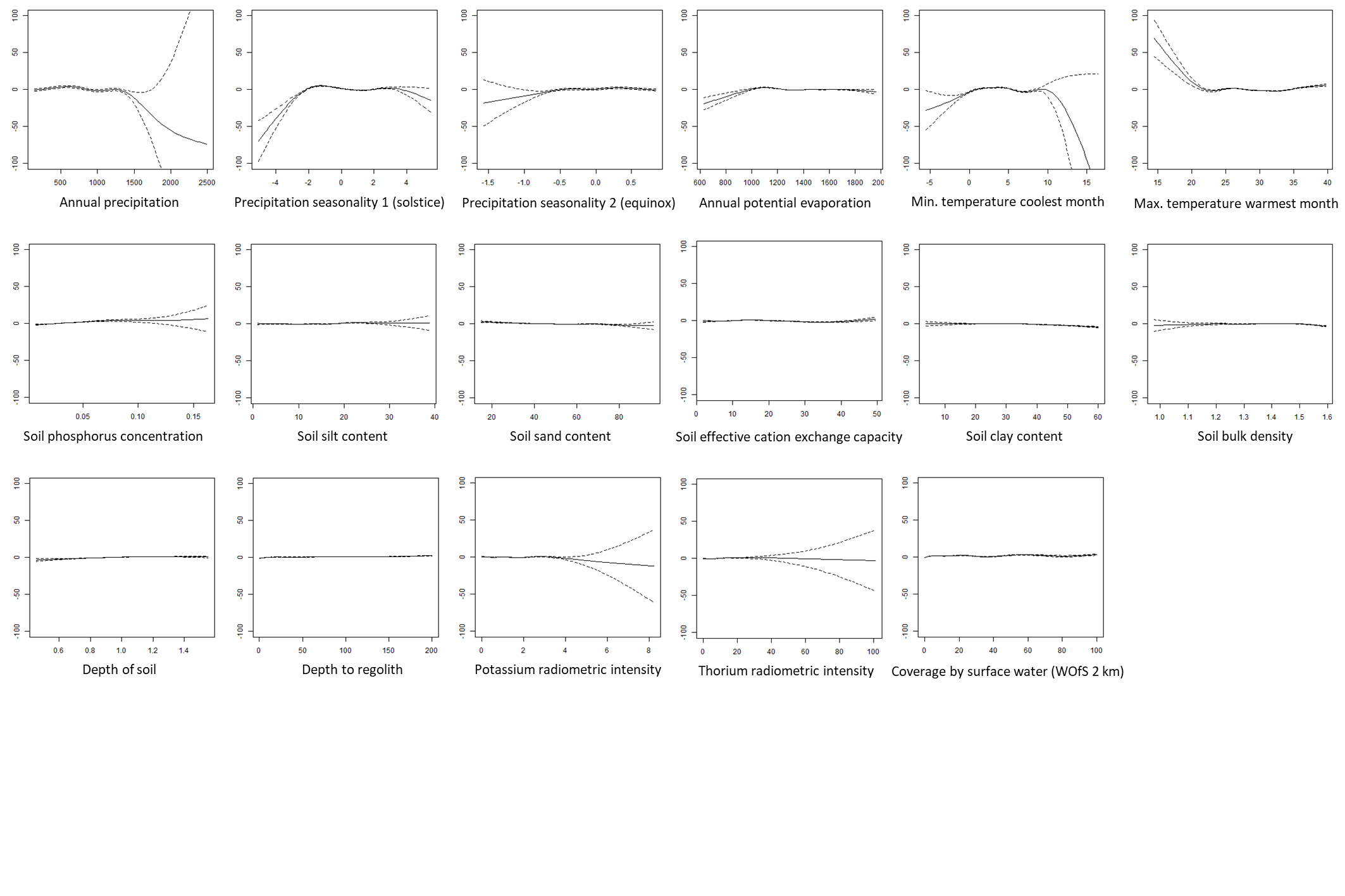


**Figure S5.1.** Model response functions for the predictor variables used in the river red gum species distribution model. The y-axis for each panel represents the model spline function for that variable. WOfS = Water Observations from Space.


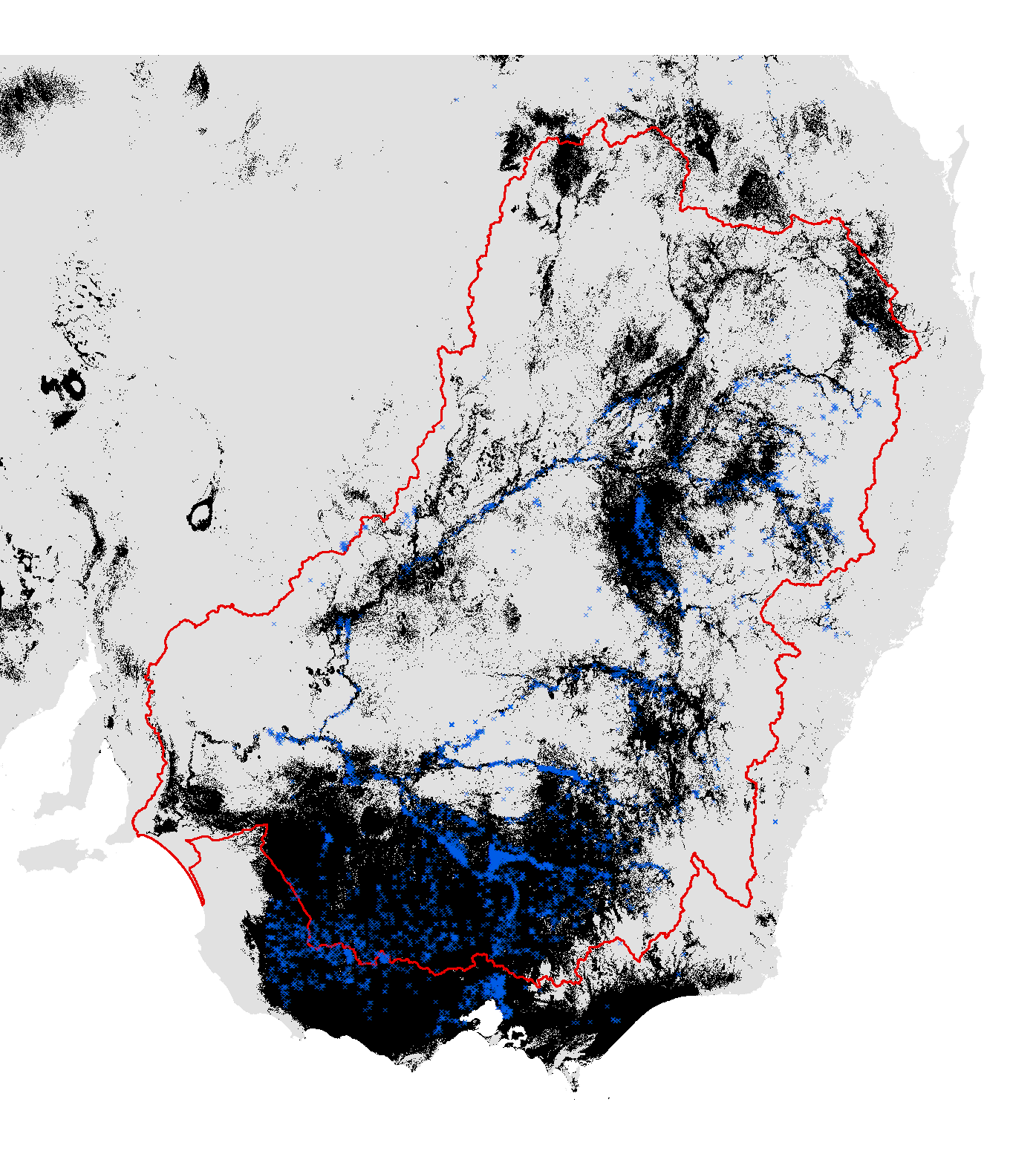


**Figure S5.2.** Predicted potential extent of occurrence for river red gum across south-east Australia (black), with observed occurrences in plant community survey plots shown in blue crosses, and the Murray-Darling Basin boundary in red.

Black box distribution model

The final model of black box potential original distribution contained 16 predictor variables and explained 61.0% deviance in black box occurrence. The strongest predictors of black box occurrence were precipitation, temperature and soil attributes (Table S5.2, Fig. S5.3). To derive a binary spatial projection of black box predicted potential extent of occurrence, we applied a threshold to the continuous predicted values, with the threshold value set to include 95% of the observed occurrence observations, to maintain a relatively generous prediction of potential original distribution (Fig. S5.4).

**Table S5.2.**  Variable contribution to the black box species distribution model for south-east Australia. The second column indicates the importance of each variable in the multi-variate model, expressed through the Chi-squared statistic, including the significance of each predictor in that model. The third column indicates the amount of deviance explained by each predictor when used independently in a single variable model, and the significance of each.

| Variable | All variable model  Chi-squared | Single variable model % deviance explained |
| --- | --- | --- |
| Coverage by surface water (WOfS maximum mean 2 km radius) | 72.9 | 7.1 |
| Soil bulk density (0–2 m depth) | 90.7 | 14.7 |
| Soil clay content (0–2 m depth) | 135.8 | 19.8 |
| Depth to regolith | 199.4 | 35.4 |
| Depth of soil | 104.8 | 12.4 |
| Soil effective cation exchange capacity (0–2 m depth) | 119.4 | 28.3 |
| Elevation focal range (300 m) | 54.7 | 24.7 |
| Soil nitrogen concentration (0–2 m depth) | 18.6 | 19.1 |
| Annual precipitation | 30.2 | 32.3 |
| Soil phosphorus concentration (0–2 m depth) | 76.7 | 4.3 |
| Precipitation seasonality 1 (solstice) | 37.6 | 18.1 |
| Precipitation seasonality 2 (equinox) | 302.2 | 11.3 |
| Soil sand content (0–2 m depth) | 102.0 | 4.3 |
| Thorium radiometric intensity | 54.6 | 3.5 |
| Minimum temperature of the coolest month | 258.5 | 20.2 |
| Maximum temperature of the warmest month | 199.0 | 20.5 |

All variables statistically significant at *P* <0.001.
WOfS = Water Observations from Space.


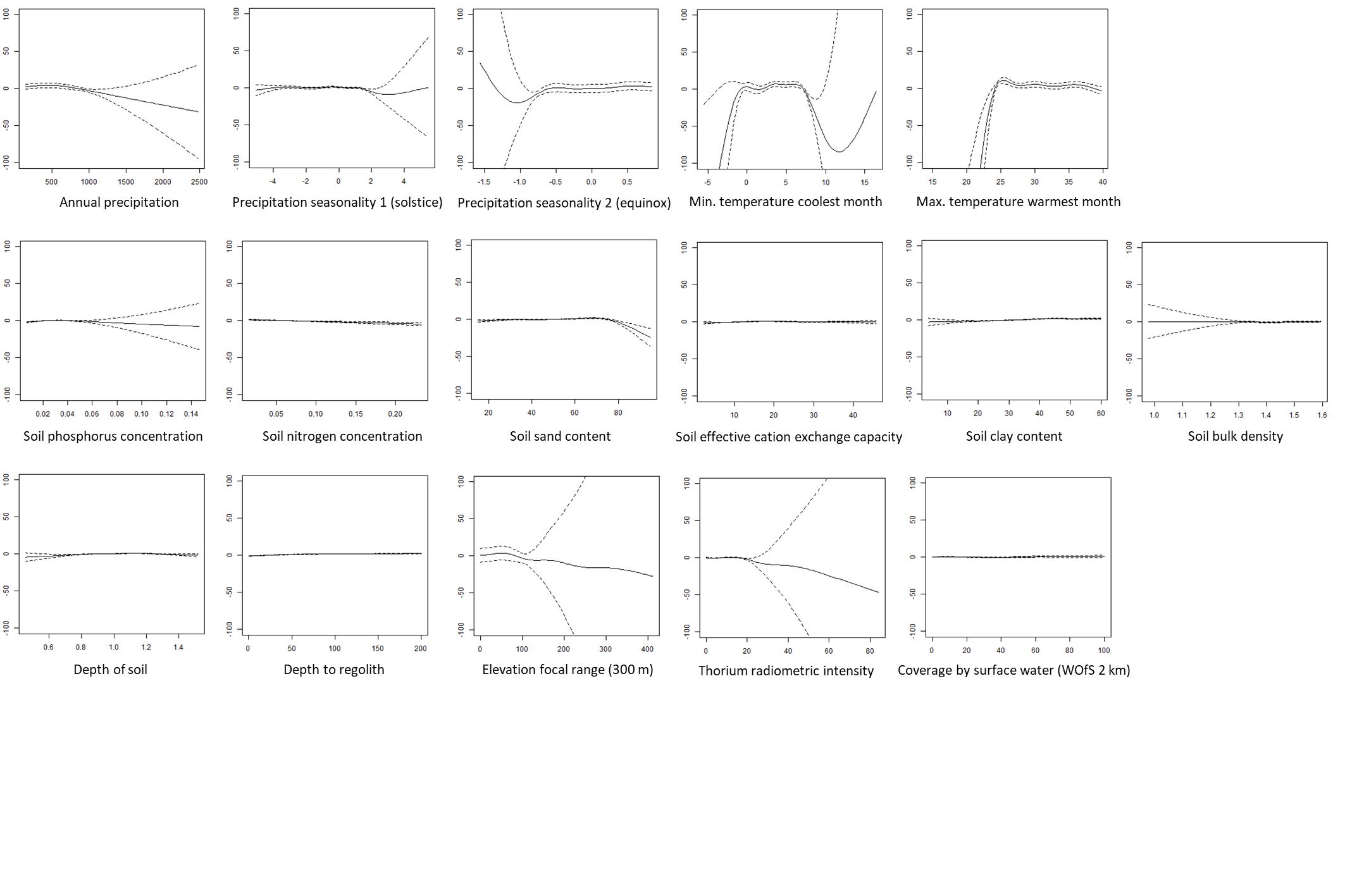


**Figure S5.3.** Model response functions for the predictor variables used in the black box species distribution model. WOfS = Water Observations from Space.


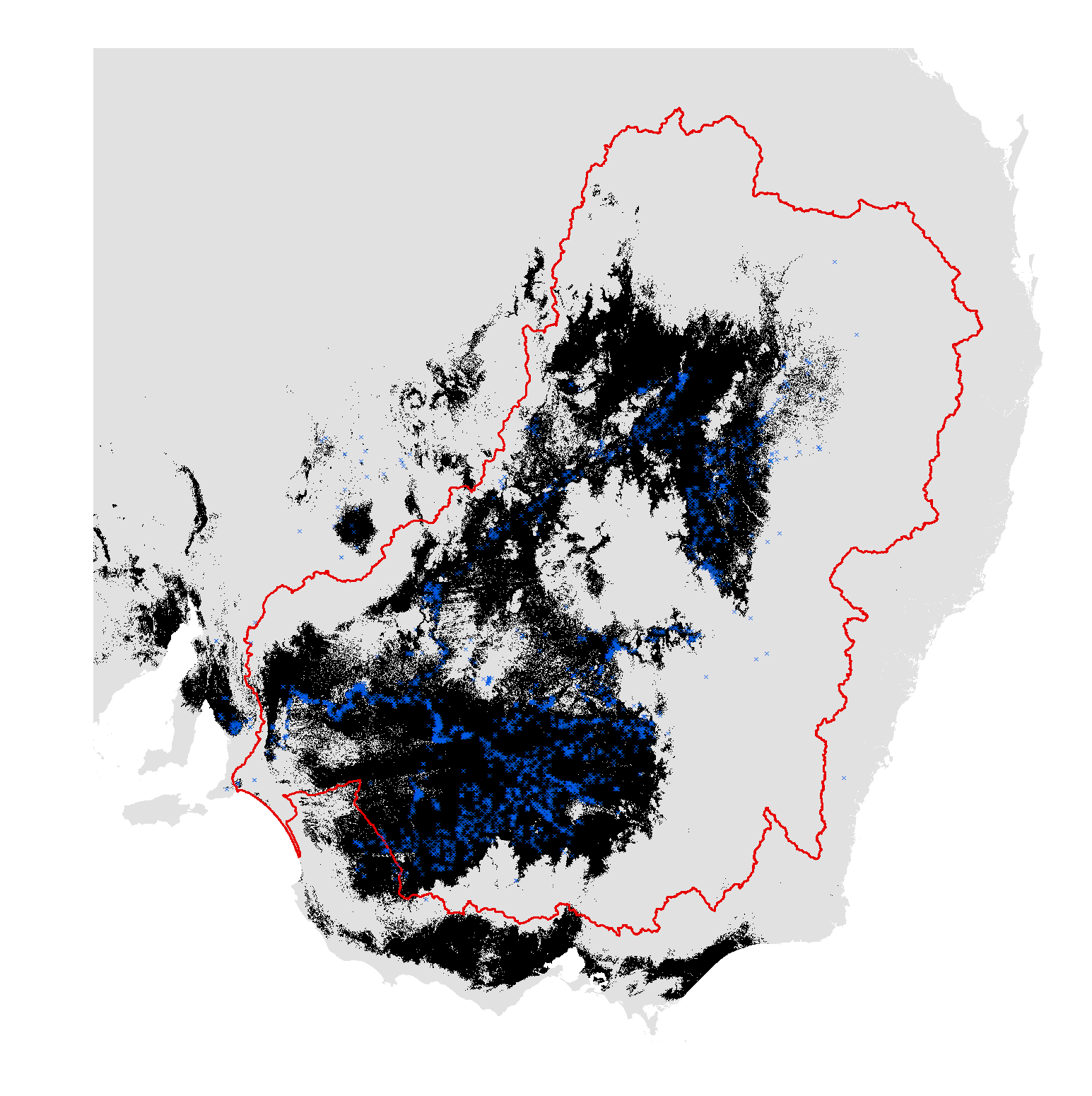


**Figure S5.4.** Predicted potential extent of occurrence for black box across south-east Australia (black), with observed occurrences in plant community survey plots shown in blue crosses, and the Murray-Darling Basin boundary in red.

**Appendix S6 - Habitat mapping for focal species**

Within the potential original distribution of each of the 10 focal species (Table S6.1), we assessed the availability of suitable habitat for the 2 years considered (2010 and 2015). To undertake this assessment, we applied the draft Land Cover Classification System (LCCS) produced by Geoscience Australia (GA 2020). We related descriptions of the habitat preferences for each species from official conservation documents in the Australian Government Species Profile and Threats Database (DAWE 2020b) to the level 3 and level 4 land cover classes (GA 2020). Intersecting the static potential extent of occurrence with the combinations of land cover classes relevant to the habitat for each species enables mapping the availability of suitable habitat for each species for each year considered (2010 and 2015) (Fig. 5). This approach is fully scalable to the continental level for Australia, providing an estimate of available habitat at fine spatial resolution (25 m). Note that due to many species having more nuanced habitat requirements that are not captured in the current land cover classes, the present approach is likely an overestimate of available habitat for the species considered here.

In contrast, several species had markedly low areas of habitat estimated based purely on the LCCS data. Specifically, these were the growling grass frog and river swamp wallaby grass, which occupy the margins of wetlands where semi-aquatic vegetation occurs. Because WOfS does not always detect standing water under vegetated areas, we augmented estimates of wetland habitat for both these species with locations of wetlands derived from the ANAE classification. Intersecting these ANAE wetlands with the other LCCS classes for these 2 species (Table S6.2), including herbaceous land cover types, potential wetland habitat for these species became restricted to shallow regions or edges of wetlands.

**Table S6.1**. Focal species assessed in species-level biodiversity accounts. Those species listed as threatened have spatial distributions indicating that they are ‘likely to occur’ in GKP (Species of National Environmental Significance (SNES) Database (DAWE 2020a)).

| Common name | Scientific name | Group | Status | Distribution | Resolution |
| --- | --- | --- | --- | --- | --- |
| Australasian bittern | *Botaurus poiciloptilus* | bird | Endangered | SNES database | 1 km |
| Painted honeyeater | *Grantiella picta* | bird | Vulnerable | SNES database | 1 km |
| Superb parrot | *Polytelis swainsonii* | bird | Vulnerable | SNES database | 10 km |
| Growling grass frog | *Litoria raniformis* | frog | Vulnerable | SNES database | 1 km |
| Koala | *Phascolarctos cinereus* | mammal | Vulnerable | SNES database | 1 km |
| Rigid spider-orchid | *Caladenia tensa* | plant | Endangered | SNES database | 10 km |
| Winged pepper-cress | *Lepidium monoplocoides* | plant | Endangered | SNES database | 1 km |
| River swamp wallaby-grass | *Amphibromus fluitans* | plant | Vulnerable | SNES database | 1 km |
| River red gum | *Eucalyptus camaldulensis* | plant | Least concern | Modelled^*^ | 90 m |
| Black box | *Eucalyptus largiflorens* | plant | Least concern | Modelled^*^ | 90 m |

*Appendix F.

**Table S6.2**. Spatial definition of available habitat for each species in relation to the draft Land Cover Classification System classes for level 3 and level 4 (lifeform, vegetation cover). The land cover attributes selected to reflect suitable habitat for each species are shown as dark shaded with ticks.

| Species | Habitat description | Level 3 landcover | | | Lifeform | | Vegetation cover | | | | |
| --- | --- | --- | --- | --- | --- | --- | --- | --- | --- | --- | --- |
|  |  | Natural terrestrial vegetation | Natural aquatic vegetation | Artificial aquatic vegetation | Woody | Herbaceous | Closed (>65%) | Open (40–65%) | Open (15–40%) | Sparse (4–15%) | Scattered (1–4%) |
| Australasian bittern  (*Botaurus poiciloptilus)* | Permanent and seasonal freshwater wetlands with tall dense vegetation dominated by sedges, rushes and reeds^*^ | 🗸 | 🗸 | 🗸 |  | 🗸 |  |  |  |  |  |
| Painted honeyeater  (*Grantiella picta)* | Eucalypt forests/woodlands containing a higher number of mature trees^*^ | 🗸 | 🗸 |  | 🗸 |  | 🗸 | 🗸 | 🗸 |  |  |
| Superb parrot (*Polytelis swainsonii)* | Riverine forests and box-gum woodlands^§^ | 🗸 | 🗸 |  | 🗸 |  | 🗸 | 🗸 | 🗸 |  |  |
| Growling grass frog  (*Litoria raniformis)* | Vegetation within or at the edges of permanent water, with emergent, submerged and floating vegetation^§^ |  | 🗸 |  |  | 🗸 |  |  |  |  |  |
| Koala  (*Phascolarctos cinereus)* | Temperate, sub-tropical and tropical forest, woodland and semi-arid communities dominated by eucalypts^*^ | 🗸 | 🗸 |  | 🗸 |  | 🗸 | 🗸 | 🗸 |  |  |
| Rigid spider-orchid  (*Caladenia tensa)* | Cypress pine, yellow gum woodland and broombush mallee on Tertiary and Quaternary aeolian sandy loams^*^ | 🗸 |  |  | 🗸 |  |  | 🗸 | 🗸 |  |  |
| Winged pepper-cress  (*Lepidium monoplocoides)* | Seasonally flooded open floodplain woodlands, grasslands & wetlands on clay soils^§^ | 🗸 | 🗸 |  | 🗸 |  |  | 🗸 | 🗸 |  |  |
| River swamp wallaby-grass (*Amphibromus fluitans)* | Natural and man-made water bodies, including swamps, lagoons, billabongs and dams^*^ |  | 🗸 |  |  | 🗸 |  |  |  |  |  |
| River red gum  (*Eucalyptus camaldulensis)* | Floodplain forest and woodlands^†^ | 🗸 | 🗸 |  | 🗸 |  | 🗸 | 🗸 | 🗸 |  |  |
| Black box  (*Eucalyptus largiflorens)* | Floodplain forest and woodlands^†^ | 🗸 | 🗸 |  | 🗸 |  | 🗸 | 🗸 | 🗸 |  |  |

^*^Species Profile and Threats Database: Conservation Advice, Department of Agriculture, Water and the Environment (DAWE 2020b).
^§^National Recovery Plan, Department of Agriculture, Water and the Environment.
^†^Newell et al. (2017).

**Appendix S7 - Biodiversity accounts for vascular plants and waterbirds.**

**Table S7.1.** Community-level biodiversity account for vascular plants and waterbirds in the Murray-Darling Basin, reported by the regions applied in the basin-wide watering strategy (MDBA 2014).

| Ecosystem accounting area | Expected plant species persistence (%) *^a^* | | | Average number of waterbird species expected *^b^* | | |
| --- | --- | --- | --- | --- | --- | --- |
|  | 2010 | 2015 | change | 2010 | 2015 | change |
| Barwon-Darling | 89.9 | 89.9 | 0 | 11.79 | 11.56 | –0.23 |
| Border Rivers | 85.6 | 85.9 | +0.3 | 11.22 | 10.98 | –0.23 |
| Campaspe | 83.1 | 83.6 | +0.5 | 11.79 | 11.69 | –0.10 |
| Condamine-Balonne | 86.7 | 86.9 | +0.2 | 13.07 | 12.40 | –0.67 |
| Eastern Mt Lofty Ranges | 83.7 | 84.1 | +0.4 | 13.04 | 13.08 | +0.04 |
| Goulburn-Broken | 84.0 | 84.5 | +0.5 | 12.16 | 12.01 | –0.14 |
| Gwydir | 85.2 | 85.4 | +0.2 | 12.36 | 12.18 | –0.18 |
| Lachlan | 86.0 | 86.3 | +0.3 | 12.17 | 12.33 | +0.17 |
| Loddon | 83.2 | 83.6 | +0.4 | 12.73 | 12.24 | –0.49 |
| Lower Darling | 90.2 | 90.3 | +0.1 | 13.41 | 12.90 | –0.51 |
| Macquarie-Castlereagh | 86.2 | 86.5 | +0.3 | 12.58 | 12.58 | 0.00 |
| Moonie | 86.6 | 86.7 | +0.1 | 13.53 | 13.21 | –0.32 |
| Murray | 86.7 | 86.9 | +0.2 | 12.49 | 12.40 | –0.10 |
| Murrumbidgee | 85.2 | 85.6 | +0.4 | 13.26 | 13.22 | –0.04 |
| Namoi | 85.1 | 85.5 | +0.4 | 11.27 | 11.01 | -0.26 |
| Nebine | 89.5 | 89.6 | +0.1 | 12.73 | 12.52 | -0.21 |
| Ovens | 84.5 | 85.0 | +0.5 | 10.63 | 10.68 | +0.04 |
| Paroo | 91.7 | 91.7 | 0 | 12.46 | 11.71 | –0.75 |
| Warrego | 89.9 | 89.9 | 0 | 12.05 | 11.75 | –0.30 |
| Wimmera-Avoca | 84.3 | 84.5 | +0.2 | 13.78 | 13.49 | –0.29 |
| All Murray-Darling Basin | 86.8 | 87.1 | +0.3 | 12.56 | 12.32 | –0.24 |

*^a^* Percentage of species originally occurring in the ecosystem accounting area that are expected to persist over the long term anywhere in their range, given changes in habitat condition across all southeastern Australia.

*^b^* Average number of species expected per ≈90-m grid cell in the ecosystem accounting area.

**Appendix S8 - Biodiversity accounts for the ten focal species.**

**Table S8.1.** Species-level biodiversity assessment in the Murray-Darling Basin, reported by the regions applied in the Basin-wide watering strategy (MDBA 2014).

| ECOSYSTEM ACCOUNTING AREA | Australasian bittern  (*Botaurus poiciloptilus*) | | | | Painted honeyeater  (*Grantiella picta*) | | | | Superb parrot  (*Polytelis swainsonii*) | | | | Growling grass frog  (*Litoria raniformis*) | | | | Koala  (*Phascolarctos cinereus*) | | | |
| --- | --- | --- | --- | --- | --- | --- | --- | --- | --- | --- | --- | --- | --- | --- | --- | --- | --- | --- | --- | --- |
|  | Potential extent of occurrence (`000 ha) | 2010 habitat (`000 ha) | 2015 habitat (`000 ha) | Change (`000 ha) | Potential extent of occurrence (`000 ha) | 2010 habitat (`000 ha) | 2015 habitat (`000 ha) | Change (`000 ha) | Potential extent of occurrence (`000 ha) | 2010 habitat (`000 ha) | 2015 habitat (`000 ha) | Change (`000 ha) | Potential extent of occurrence (`000 ha) | 2010 habitat (`000 ha) | 2015 habitat (`000 ha) | Change (`000 ha) | Potential extent of occurrence (`000 ha) | 2010 habitat (`000 ha) | 2015 habitat (`000 ha) | Change (`000 ha) |
| Barwon-Darling | 859.63 | 0.40 | 0.40 | 0.00 | 4311.25 | 967.23 | 1546.10 | +578.87 | 920.17 | 299.55 | 354.66 | +55.11 | 0.00 | 0.00 | 0.00 | 0.00 | 1694.29 | 368.95 | 506.73 | +137.78 |
| Border Rivers | 1615.59 | 3.04 | 3.04 | 0.00 | 4567.45 | 1437.28 | 1648.93 | +211.65 | 0.00 | 0.00 | 0.00 | 0.00 | 0.00 | 0.00 | 0.00 | 0.00 | 4567.45 | 1437.28 | 1648.93 | +211.65 |
| Campaspe | 408.37 | 0.01 | 0.01 | 0.00 | 427.88 | 103.62 | 84.45 | –19.17 | 92.31 | 2.87 | 2.09 | –0.78 | 427.88 | 0.01 | 0.01 | 0.00 | 427.88 | 103.62 | 84.45 | –19.17 |
| Condamine-Balonne | 1490.09 | 0.91 | 0.91 | 0.00 | 11200.77 | 2521.02 | 2386.74 | –134.28 | 1.90 | 0.02 | 0.00 | –0.02 | 0.00 | 0.00 | 0.00 | 0.00 | 11071.6 | 2505.71 | 2372.93 | –132.78 |
| Eastern Mt Lofty Ranges | 320.63 | 0.62 | 0.61 | –0.01 | 356.79 | 25.28 | 30.74 | +5.46 | 0.00 | 0.00 | 0.00 | 0.00 | 157.51 | 0.51 | 0.50 | –0.01 | 352.92 | 25.23 | 30.52 | +5.29 |
| Goulburn-Broken | 1826.92 | 1.50 | 1.50 | 0.00 | 1933.64 | 616.09 | 568.50 | –47.59 | 601.62 | 48.68 | 50.29 | +1.61 | 2183.11 | 1.55 | 1.55 | 0.00 | 2183.11 | 850.14 | 800.55 | –49.59 |
| Gwydir | 1138.19 | 0.67 | 0.67 | 0.00 | 2899.76 | 560.07 | 691.00 | +130.93 | 255.82 | 36.46 | 36.17 | –0.30 | 0.00 | 0.00 | 0.00 | 0.00 | 2899.76 | 560.07 | 691.00 | +130.93 |
| Lachlan | 6684.13 | 3.22 | 3.22 | 0.00 | 8652.92 | 1169.18 | 1533.72 | +364.53 | 6178.12 | 943.43 | 1142.84 | +199.41 | 601.08 | 0.30 | 0.30 | 0.00 | 8655.39 | 1170.68 | 1535.92 | +365.24 |
| Loddon | 1314.86 | 0.20 | 0.20 | 0.00 | 1332.11 | 260.60 | 217.98 | -42.62 | 414.62 | 11.52 | 6.76 | –4.77 | 1332.11 | 0.20 | 0.20 | 0.00 | 1332.11 | 260.60 | 217.98 | –42.62 |
| Lower Darling | 1421.84 | 4.82 | 4.82 | 0.00 | 5584.08 | 64.38 | 338.69 | +274.30 | 0.00 | 0.00 | 0.00 | 0.00 | 268.10 | 4.56 | 4.56 | 0.00 | 2407.80 | 55.97 | 279.04 | +223.07 |
| Macquarie-Castlereagh | 6170.13 | 20.35 | 20.35 | 0.00 | 9175.27 | 1954.55 | 1746.57 | -207.98 | 7694.54 | 1597.56 | 1432.35 | –165.21 | 0.00 | 0.00 | 0.00 | 0.00 | 9085.53 | 1911.08 | 1696.16 | –214.92 |
| Moonie | 325.70 | 0.35 | 0.35 | 0.00 | 1510.32 | 248.89 | 224.46 | -24.43 | 0.00 | 0.00 | 0.00 | 0.00 | 0.00 | 0.00 | 0.00 | 0.00 | 1510.32 | 248.89 | 224.46 | –24.43 |
| Murray | 4873.09 | 29.36 | 29.66 | +0.30 | 8574.37 | 960.10 | 1189.40 | +229.30 | 1861.48 | 294.61 | 274.03 | –20.58 | 4184.06 | 25.72 | 26.03 | +0.31 | 6988.49 | 1461.11 | 1600.42 | +139.31 |
| Murrumbidgee | 7192.82 | 8.65 | 8.65 | 0.00 | 8299.97 | 871.17 | 1127.29 | +256.13 | 7075.53 | 856.35 | 1048.03 | +191.68 | 5545.35 | 8.64 | 8.64 | 0.00 | 8779.50 | 1288.48 | 1568.09 | +279.60 |
| Namoi | 1697.01 | 11.68 | 11.68 | 0.00 | 4180.27 | 1505.24 | 1460.25 | –44.99 | 1433.18 | 701.79 | 631.11 | –70.69 | 0.00 | 0.00 | 0.00 | 0.00 | 4185.57 | 1507.49 | 1462.66 | –44.83 |
| Nebine | 2.97 | 0.00 | 0.00 | 0.00 | 3809.35 | 496.37 | 211.43 | –284.94 | 0.00 | 0.00 | 0.00 | 0.00 | 0.00 | 0.00 | 0.00 | 0.00 | 3809.35 | 496.37 | 211.43 | –284.94 |
| Ovens | 430.38 | 0.46 | 0.46 | 0.00 | 594.74 | 252.16 | 276.22 | +24.06 | 222.03 | 42.17 | 53.26 | +11.10 | 690.11 | 0.62 | 0.62 | 0.00 | 786.71 | 440.75 | 466.13 | +25.37 |
| Paroo | 6.29 | 0.00 | 0.00 | 0.00 | 5531.38 | 166.26 | 202.88 | +36.62 | 0.00 | 0.00 | 0.00 | 0.00 | 0.00 | 0.00 | 0.00 | 0.00 | 832.51 | 96.51 | 28.69 | –67.82 |
| Warrego | 51.72 | 0.03 | 0.03 | 0.00 | 7798.15 | 1511.70 | 1051.99 | –459.71 | 0.00 | 0.00 | 0.00 | 0.00 | 0.00 | 0.00 | 0.00 | 0.00 | 6371.98 | 1441.23 | 859.74 | –581.49 |
| Wimmera-Avoca | 4417.25 | 4.46 | 4.46 | 0.00 | 4422.66 | 219.80 | 247.92 | +28.12 | 0.00 | 0.00 | 0.00 | 0.00 | 3640.81 | 3.85 | 3.85 | 0.00 | 3074.47 | 213.68 | 220.94 | +7.26 |
| All Murray-Darling Basin | 42247.6 | 90.70 | 91.00 | +0.30 | 95163.14 | 15910.97 | 16785.25 | +874.28 | 26751.3 | 4835.0 | 5031.58 | +196.58 | 19030.1 | 45.96 | 46.27 | +0.31 | 81016.7 | 16443.8 | 16506.7 | +62.91 |

Table S7.1 (cont.)

| ECOSYSTEM ACCOUNTING AREA | RIGID SPIDER-ORCHID  (*Caladenia tensa*) | | | | Winged pepper-cress  (*Lepidium monoplocoides*) | | | | River swamp wallaby-grass  (*Amphibromus fluitans*) | | | | River red gum  (*Eucalyptus camaldulensis*) | | | | Black box  (*Eucalyptus largiflorens*) | | | |
| --- | --- | --- | --- | --- | --- | --- | --- | --- | --- | --- | --- | --- | --- | --- | --- | --- | --- | --- | --- | --- |
|  | Potential extent of occurrence (`000 ha) | 2010 habitat (`000 ha) | 2015 habitat (`000 ha) | Change (`000 ha) | Potential extent of occurrence (`000 ha) | 2010 habitat (`000 ha) | 2015 habitat (`000 ha) | Change (`000 ha) | Potential extent of occurrence (`000 ha) | 2010 habitat (`000 ha) | 2015 habitat (`000 ha) | Change (`000 ha) | Potential extent of occurrence (`000 ha) | 2010 habitat (`000 ha) | 2015 habitat (`000 ha) | Change (`000 ha) | Potential extent of occurrence (`000 ha) | 2010 habitat (`000 ha) | 2015 habitat (`000 ha) | Change (`000 ha) |
| Barwon-Darling | 0.00 | 0.00 | 0.00 | 0.00 | 1227.83 | 378.61 | 566.26 | +187.65 | 0.00 | 0.00 | 0.00 | 0.00 | 565.29 | 54.59 | 76.30 | +21.71 | 1581.00 | 297.60 | 452.14 | +154.55 |
| Border Rivers | 0.00 | 0.00 | 0.00 | 0.00 | 1.73 | 0.06 | 0.12 | +0.06 | 0.00 | 0.00 | 0.00 | 0.00 | 449.95 | 77.21 | 85.79 | +8.58 | 104.40 | 5.87 | 5.45 | –0.41 |
| Campaspe | 276.26 | 64.69 | 73.06 | +8.37 | 0.00 | 0.00 | 0.00 | 0.00 | 413.09 | 0.01 | 0.01 | 0.00 | 427.54 | 103.42 | 84.22 | –19.21 | 119.90 | 4.29 | 3.11 | –1.18 |
| Condamine-Balonne | 0.00 | 0.00 | 0.00 | 0.00 | 3.43 | 0.99 | 0.09 | –0.90 | 0.00 | 0.00 | 0.00 | 0.00 | 3572.61 | 471.16 | 398.67 | –72.49 | 2731.63 | 148.32 | 31.97 | –116.35 |
| Eastern Mt Lofty Ranges | 361.09 | 21.50 | 29.43 | +7.92 | 0.00 | 0.00 | 0.00 | 0.00 | 0.00 | 0.00 | 0.00 | 0.00 | 102.85 | 1.87 | 3.96 | +2.09 | 67.88 | 1.63 | 2.67 | +1.05 |
| Goulburn-Broken | 186.05 | 59.55 | 58.88 | –0.67 | 0.00 | 0.00 | 0.00 | 0.00 | 1872.53 | 1.50 | 1.50 | 0.00 | 1988.02 | 655.23 | 607.60 | –47.62 | 565.69 | 49.47 | 43.35 | –6.12 |
| Gwydir | 0.00 | 0.00 | 0.00 | 0.00 | 0.00 | 0.00 | 0.00 | 0.00 | 0.00 | 0.00 | 0.00 | 0.00 | 767.18 | 47.74 | 57.27 | +9.54 | 259.80 | 5.39 | 4.79 | –0.60 |
| Lachlan | 402.44 | 53.58 | 61.69 | +8.11 | 2213.90 | 45.84 | 51.40 | +5.55 | 370.48 | 0.00 | 0.00 | 0.00 | 2066.85 | 178.91 | 220.87 | +41.96 | 2143.43 | 102.76 | 142.54 | +39.78 |
| Loddon | 634.98 | 170.70 | 155.55 | –15.14 | 106.10 | 2.08 | 1.47 | –0.61 | 1310.92 | 0.20 | 0.20 | 0.00 | 1329.17 | 259.97 | 217.40 | –42.57 | 636.50 | 19.86 | 10.70 | –9.16 |
| Lower Darling | 138.63 | 7.73 | 10.82 | +3.09 | 2758.70 | 20.32 | 152.88 | 132.56 | 0.00 | 0.00 | 0.00 | 0.00 | 1712.16 | 28.68 | 87.15 | +58.47 | 5124.09 | 52.17 | 226.73 | +174.56 |
| Macquarie-Castlereagh | 0.00 | 0.00 | 0.00 | 0.00 | 62.95 | 30.78 | 30.47 | –0.32 | 0.00 | 0.00 | 0.00 | 0.00 | 3282.44 | 352.28 | 168.16 | –184.12 | 3459.19 | 331.29 | 174.63 | –156.66 |
| Moonie | 0.00 | 0.00 | 0.00 | 0.00 | 0.00 | 0.00 | 0.00 | 0.00 | 0.00 | 0.00 | 0.00 | 0.00 | 178.06 | 15.02 | 10.87 | –4.14 | 150.17 | 6.71 | 3.18 | –3.53 |
| Murray | 6483.66 | 258.66 | 517.18 | +258.52 | 1072.23 | 71.27 | 93.36 | +22.09 | 2324.23 | 4.89 | 4.89 | 0.00 | 4882.31 | 468.03 | 582.32 | +114.29 | 7145.26 | 208.37 | 392.52 | +184.15 |
| Murrumbidgee | 284.68 | 33.02 | 40.18 | +7.17 | 2435.90 | 51.74 | 75.05 | +23.30 | 1848.40 | 0.54 | 0.54 | 0.00 | 3221.36 | 168.47 | 248.05 | +79.58 | 3600.06 | 77.17 | 123.35 | +46.18 |
| Namoi | 0.00 | 0.00 | 0.00 | 0.00 | 0.00 | 0.00 | 0.00 | 0.00 | 0.00 | 0.00 | 0.00 | 0.00 | 1331.38 | 271.29 | 224.90 | –46.39 | 290.37 | 13.51 | 1.08 | –12.44 |
| Nebine | 0.00 | 0.00 | 0.00 | 0.00 | 0.00 | 0.00 | 0.00 | 0.00 | 0.00 | 0.00 | 0.00 | 0.00 | 590.61 | 32.86 | 4.37 | –28.49 | 950.58 | 50.55 | 11.48 | –39.07 |
| Ovens | 31.56 | 1.96 | 3.22 | +1.26 | 0.00 | 0.00 | 0.00 | 0.00 | 437.28 | 0.46 | 0.46 | 0.00 | 632.16 | 285.34 | 308.33 | +22.99 | 41.93 | 3.33 | 4.15 | +0.81 |
| Paroo | 0.00 | 0.00 | 0.00 | 0.00 | 0.00 | 0.00 | 0.00 | 0.00 | 0.00 | 0.00 | 0.00 | 0.00 | 654.08 | 14.20 | 5.34 | –8.87 | 2186.99 | 39.12 | 80.04 | +40.92 |
| Warrego | 0.00 | 0.00 | 0.00 | 0.00 | 0.00 | 0.00 | 0.00 | 0.00 | 0.00 | 0.00 | 0.00 | 0.00 | 1450.23 | 129.74 | 64.93 | –64.81 | 3177.12 | 180.17 | 175.92 | –4.25 |
| Wimmera-Avoca | 4141.96 | 186.66 | 220.84 | +34.18 | 253.80 | 3.95 | 9.64 | +5.69 | 1371.93 | 2.30 | 2.30 | 0.00 | 4132.80 | 216.08 | 240.68 | +24.60 | 3650.11 | 25.85 | 76.40 | +50.55 |
| All Murray-Darling Basin | 12941.28 | 858.05 | 1170.87 | +312.82 | 10136.57 | 605.66 | 980.73 | +375.08 | 9948.83 | 9.90 | 9.90 | 0.00 | 33337.1 | 3832.09 | 3697.16 | –134.92 | 37986.1 | 1623.44 | 1966.23 | +342.78 |

**References**

BOM (2012). Australian Hydrological Geospatial Fabric (Geofabric). Version 2.1. Commonwealth of Australia (Bureau of Meteorology). In.

Brooks, S. (2017). Classification of aquatic ecosystems in the Murray-Darling Basin: 2017 update. Report to the Murray-Darling Basin Authority and Commonwealth Environmental Water Office, Canberra. In.

Brooks, S., Cottingham, P., Butcher, R. & J., H. (2014). Murray-Darling Basin aquatic ecosystem classification: Stage 2 report. Peter Cottingham & Associates report to the Commonwealth Environmental Water Office and Murray-Darling Basin Authority, Canberra. In.

DAWE (2020a). Australia - Species of National Environmental Significance Distributions (public grids). Commonwealth of Australia (Department of Agriculture, Water and the Environment). In.

DAWE (2020b). Species Profile and Threats Database (SPRAT). URL <http://www.environment.gov.au/cgi-bin/sprat/public/sprat.pl>

Evans, J.P., Ji, F., Lee, C., Smith, P., Argüeso, D. & Fita, L. (2014). Design of a regional climate modelling projection ensemble experiment – NARCliM. *Geosci. Model Dev.*, 7, 621–629.

Ferrier, S., Manion, G., Elith, J. & Richardson, K. (2007). Using generalized dissimilarity modelling to analyse and predict patterns of beta diversity in regional biodiversity assessment. *Diversity and Distributions*, 13, 252-264.

Ferrier, S., Powell, G.V.N., Richardson, K.S., Manion, G., Overton, J.M., Allnutt, T.F. *et al.* (2004). Mapping more of terrestrial biodiversity for global conservation assessment. *Bioscience*, 54, 1101-1109.

GA (2020). Landcover Classification System for Australia (2010 and 2015): Draft version 0.5. Geoscience Australia. In: (ed. Australia G). Geoscience Australia Canberra.

Gallant, J. & Austin, J.M. (2012a). Relief - Elevation Range over 300 m derived from 1" SRTM DEM-S. v2. CSIRO. Data Collection. In.

Gallant, J. & Austin, J.M. (2012b). Topographic Wetness Index derived from 1" SRTM DEM-H. v2. CSIRO. Data Collection. In.

Gallant, J., Wilson, N., Dowling, T., Read, A. & Inskeep, C. (2011). SRTM-derived 3 Second Digital Elevation Models Version 1.0. Geoscience Australia, Commonwealth of Australia, Canberra.

Gallant, J.C. & Austin, J.M. (2015). Derivation of terrain covariates for digital soil mapping in Australia. *Soil Research*, 53, 895-906.

Grundy, M., Viscarra Rossel, R.A., Searle, R., Wilson, P., Chen, C. & Gregory, L. (2015). Soil and Landscape Grid of Australia. *Soil Research*, 53, 835-844.

Harwood, T.D., King, D., Nolan, M., Gallant, J., Ware, C., Austin, J. *et al.* (2018). 3 second abiotic environmental raster data for the NARCLIM region of Australia aggregated from various sources for modelling biodiversity patterns. v2. CSIRO Data Collection. In: (ed. CSIRO). CSIRO. Data Collection. CSIRO.

Harwood, T.D., Richards, A.E., Williams, K.J., Mokany, K., Schmidt, R., Ware, C. *et al.* (2021). Assessing condition of ecosystem types at Gunbower-Koondrook-Perricoota Forest Icon Site. A technical report for the Land and Ecosystem Accounts Project. In. CSIRO Canberra, Australia.

Hastie, T. & Tibshirani, R. (1986). Generalized Additive Models. *Statistical Science*, 1, 297-310.

MacArthur, R.H. (1965). Patterns of species diversity. *Biological Reviews*, 40, 510-533.

Manion, G., Lisk, M., Ferrier, S., Nieto-Lugilde, D., Mokany, K. & Fitzpatrick, M.C. (2018). gdm: Generalized Dissimilarity Modeling. In. 'gdm' R package version 1.3.7.

Mokany, K., Harwood, T.D. & Ferrier, S. (2019). Improving links between environmental accounting and scenario-based cumulative impact assessment for better-informed biodiversity decisions. *Journal of Applied Ecology*, 56, 2732-2741.

Mokany, K., Harwood, T.D., Ware, C., Williams, K.J., King, D., Nolan, M. *et al.* (2018). Enhancing landscape data: capacity building for GDM analyses to support biodiversity assessment. In. CSIRO Canberra, p. 77.

Mokany, K., Jones, M.M. & Harwood, T.D. (2013). Scaling pairwise β-diversity and α-diversity with area. *Journal of Biogeography*, 40, 2299-2309.

Mokany, K., Westcott, D.A., Prasad, S., Ford, A.J. & Metcalfe, D.J. (2014). Identifying priority areas for conservation and management in diverse tropical forests. *PLoS ONE*, 9, e89084.

Mueller, N., Lewis, A., Roberts, D., Ring, S., Melrose, R., Sixsmith, J. *et al.* (2016). Water observations from space: Mapping surface water from 25 years of Landsat imagery across Australia. *Remote Sensing of Environment*, 174, 341-352.

Newell, G., White, M. & Griffioen, P. (2017). Development of a Stand Condition Monitoring Tool for the Murray Darling Basin. In. Murray‒Darling Basin Authority Canberra, Australia.

R Development Core Team (2020). R: A Language and Environment for Statistical Computing. In. R Foundation for Statistical Computing Vienna, Austria.

Raymond, B., VanDerWal, J., L., B., Sumner, M., August, T. & Baumgartner, J. (2017). ALA4R: Atlas of Living Australia (ALA) Data and Resources in R. R package version 1.5.6. In.

Reid, J.R.W., Colloff, M.J., Arthur, A.D. & McGinness, H.M. (2013). Influence of Catchment Condition and water resource development on waterbird assemblages in the Murray-Darling Basin, Australia. *Biological Conservation*, 165, 25-34.

Reside, A., VanDerWal, J., Phillips, B., Shoo, L.P., Rosauer, D., Anderson, B. *et al.* (2013). Climate change refugia for terrestrial biodiversity: Defining areas that promote species persistence and ecosystem resilience in the face of global climate change. In. National Climate Change Adaptation Research Facility, Griffith University Gold Coast, QLD, Australia.

Rosenzweig, M.L. (1995). *Species Diversity in Space and Time*. Cambridge University Press, Cambridge.

Sørensen, T. (1948). A method of establishing groups of equal amplitude in plant sociology based on similarity of species and its application to analyses of the vegetation on Danish commons. *Biologiske Skrifter / Kongelige Danske Videnskabernes Selskab*, 5, 1-34.

Viscarra Rossel, R.A., Chen, C., Grundy, M.J., Searle, R., Clifford, D. & Campbell, P.H. (2015). The Australian three-dimensional soil grid: Australia’s contribution to the GlobalSoilMap project. *Soil Research*, 53, 845-864.

Williams, K.J., Belbin, L., Austin, M.P., Stein, J.L. & Ferrier, S. (2012). Which environmental variables should I use in my biodiversity model? *Int. J. Geogr. Inf. Sci.*, 26, 2009-2047.

Williams, K.J., Harwood, T.D., Lehmann, E.A., Ware, C., Lyon, P., Bakar, S. *et al.* (2021). Habitat Condition Assessment System (HCAS version 2.1): Enhanced method for mapping habitat condition and change across Australia. In. CSIRO Australia.

Wood, S. (2016). Mixed GAM Computation Vehicle with GCV/AIC/REML Smoothness Estimation. In. 'mgcv' R package.

Xu, T. & Hutchinson, M. (2010). ANUClim Version 6.1 User Guide. In. Fenner School of Environment and Society, The Australian National University Canberra.

1. Records accessed through ALA website and provided by: Australian Museum, Australian Museum Ornithology Collection, Australian National Wildlife Collection, BioCollect, BioNet, BirdLife Australia, Birds Australia, Citizen Science - ALA Website, Commonwealth Scientific and Industrial Research Organisation, FieldData / Biological Data Recording System, Flickr, Gaia Guide, Museums Victoria, Museums Victoria Ornithology Collection, NatureMap, NatureShare, NSW Bird Atlassers, Ocean Biogeographic Information System, Office of Environment and Heritage, OZCAM (Online Zoological Collections of Australian Museums) Provider, Queen Victoria Museum and Art Gallery, Queen Victoria Museum and Art Gallery - Birds, Queensland Department of Environment and Resource Management, Queensland Museum, Queensland Museum Birds, Questagame, South Australian Museum, South Australian Museum Ornithology Collection, Victorian Biodiversity Atlas, WCPS Bird Survey Project. [↑](#footnote-ref-1)
